# Traditional Chinese exercise in chronic obstructive pulmonary disease: An overview of systematic reviews

**DOI:** 10.1097/MD.0000000000038700

**Published:** 2024-06-28

**Authors:** Lu Han, Jing Wang, Yin Zhu, Ying Lu, Chaoqun Liu, Chaoyang Chen, Jie Li

**Affiliations:** a Shanghai Qigong Research Institute, Shanghai, China; b Taiji Health Center, Shanghai University of T.C.M/Shanghai Academy of T.C.M., Shanghai, China; c Shanghai Baoshan District Hospital of Integrated Traditional and Western Medicine, Shanghai, China; d Shanghai University of Traditional Chinese Medicine, Shanghai, China.

**Keywords:** chronic obstructive pulmonary disease, overview of systematic reviews, traditional Chinese exercise

## Abstract

This study aims to critically reassess existing systematic reviews (SR) on Traditional Chinese Exercises (TCE) for treating Chronic Obstructive Pulmonary Disease (COPD). The primary objectives include synthesizing available evidence, evaluating the methodological quality of reviews and overall evidence, and providing comprehensive insights into the effectiveness of different TCE types in managing COPD. Sinomed, CNKI, VIP, Wanfang, PubMed, Cochrane Library, and Web of Science were searched from inception to April 2023 for SR literature on the treatment of COPD with TCE. The extracted data from the included SRs encompassed various aspects such as general information, study population, intervention measures, meta-analysis results, and conclusions. The methodological quality of the included SRs was assessed using the AMSTAR II tool. Additionally, the GRADE tool was used to determine the evidence level of outcome indicators. This study included 17 SRs and 4 types of TCE. The CCA was 0.041, indicating a slight overlap between the primary studies. Notably, one study was rated as low quality on the AMSTAR II scale, while the rest were classified as critically low quality. The results from the GRADE evaluation revealed 26 pieces of very low-quality evidence, 55 pieces of low-quality evidence, and 17 pieces of moderate-quality evidence. The moderate-quality evidence suggests that Liuzijue effectively improves TCM syndrome scores in patients with COPD. Additionally, low-quality evidence suggests that Liuzijue improves patients’ lung function (FEV1, FVC) and quality of life (CAT, MRC/mMRC). Similarly, low-quality evidence suggests that Baduanjin can improve patients’ lung function (FEV1%, FVC) and quality of life (SGRQ). Low-quality evidence also suggests that Health Qigong can significantly improve patients’ exercise endurance (6MWD). No SR reported TCE-related adverse reactions. TCE interventions are effective and safe in the treatment of COPD. Different types of TCE have varying effects on outcomes in COPD patients. However, these findings are limited by the generally low methodological and evidence quality of the included SRs. Therefore, it is strongly recommended to improve study designs to obtain higher-quality clinical evidence and to strictly follow SR protocols.

## 1. Introduction

Chronic Obstructive Pulmonary Disease (COPD) is a heterogeneous pulmonary condition characterized by chronic respiratory symptoms (such as dyspnea, cough, and sputum production) and persistent (often progressive) airflow obstruction. It results from airway abnormalities (such as chronic bronchitis and small airway inflammation) and/or alveolar abnormalities (emphysema).^[1]^ Given its high prevalence, disability burden, mortality rate, and overall disease burden, COPD has emerged as a pressing global public health concern. Recent data indicates that COPD is the third leading cause of death globally and the seventh leading contributor to poor health, as measured by Disability-Adjusted Life Years (DALYs).^[2,3]^ In 2019, COPD accounted for 3.23 million deaths worldwide, and this number is projected to significantly increase in the coming decades due to aging populations and increasing air pollution. The World Health Organization (WHO) estimates that by 2060, the annual deaths attributable to COPD and related diseases will exceed 5.4 million. A comprehensive analysis of the disease burden revealed that COPD imposes the greatest medical expenses on impoverished populations, and early diagnosis is crucial. The 2023 Global Initiative for Chronic Obstructive Lung Disease (GOLD) guideline recommends the implementation of COPD screening tools, including risk factor assessment, symptom evaluation, healthcare resource utilization, and simple peak expiratory flow measurements, particularly in low- and middle-income countries, to facilitate early COPD diagnosis.^[4]^

Although COPD is incurable, pulmonary rehabilitation has emerged as a pivotal therapeutic approach, with exercise rehabilitation being a cornerstone of this strategy.^[5–8]^ Traditional Chinese Exercise (TCE), rooted in the ancient traditions of Chinese medicine, encompasses a combination of physical exercises, breathing techniques, and mental focus.^[9]^ TCE have been extensively applied as a therapeutic modality, dating back to the Qin and Han dynasties, and have been historically associated with the management of respiratory ailments.^[10–12]^ Furthermore, previous studies have demonstrated that TCE modulates the immune system and enhances respiratory muscle strength, resulting in reduced dyspnea, improved exercise tolerance, and enhanced quality of life among patients with COPD.^[13–17]^ Although systematic reviews (SRs) have supported the potential benefits of TCE for patients with COPD,^[18]^ the methodological quality of these SRs is still unclear, and SRs on the same topic may arrive at conflicting conclusions due to the use of different evaluation methods, inclusion and exclusion criteria, outcome indicators, etc., which may lead to potential clinical decision-making biases.

This study conducts a re-analysis of existing SRs to comprehensively assess the efficacy and safety of diverse TCE interventions for COPD treatment. The methodological quality and reliability of conclusions in the included SRs were assessed using established tools for quality evaluation and evidence grading systems. The study provides evidence-based insights and clinical guidance from multiple perspectives to assist in making well-informed choices rooted in robust evidence and clinical reference, thereby enhancing the quality of COPD care and treatment outcomes.

## 2. Methods

### 2.1. Literature search

China National Knowledge Infrastructure (CNKI), Chinese Biomedical Literature Service System (SinoMed), VIP Database for Chinese Technical Periodicals (VIP), Wanfang Database, PubMed, Cochrane Library, and Web of Science were comprehensively searched from inception to April 2023. The search strategy employed a combination of free terms and controlled vocabulary terms. Additionally, the references of identified articles were traced to supplement the database search for potentially overlooked SRs.

The search terms included (“COPD” OR “Chronic Obstructive Pulmonary Disease”) AND (“qigong” OR “qi-training” OR “Chi Kung” OR “qi chung” OR “chi chung” OR “chi gong” OR “wuqinxi” OR “five-animal play” OR “baduanjin” OR “eight-section brocade” OR “yijinjing” OR “liuzijue” OR “daoyin”) AND (“systematic review” OR “Meta-Analysis”).

### 2.2. Study selection

Studies must meet the following criteria for inclusion: Study Subjects: The included study must involve patients diagnosed with COPD according to clinically recognized diagnostic criteria from either Western or traditional Chinese medicine. Intervention Measures: Included studies must involve only TCE, including Wuqinxi, Baduanjin, Liuzijue, Yijinjing, or different types of unspecified TCE; The included studies must involve the use of TCE combined with medication or non-pharmacological therapies. Outcome Indicators: Primary outcome indicators: Lung function – Forced expiratory volume in 1 second (FEV1), Forced vital capacity (FVC), FEV1 as a percentage of predicted value (FEV1%), FEV1/FVC ratio (FEV1/FVC %); Exercise endurance – 6-minute walk distance (6MWD); Quality of life – COPD Assessment Test (CAT); Modified Medical Research Council Dyspnea Scale (MRC/mMRC); St. George’s Respiratory Questionnaire (SGRQ); Secondary outcome indicators: Traditional Chinese medicine (TCM) symptom scale; Adverse events. A study was included if any of these indicators were reported. Study Type: Interventional SR literature, published in either Chinese or English.

The following studies were excluded: Studies involving COPD patients with other diseases, regardless of the specific comorbidity. SRs that use external qigong, meditation, or yoga as intervention measures. SRs involving complex interventions where the effects of TCE cannot be determined. Notably, some SRs involved TCE, alongside aerobic exercise and suspension training, making it difficult to ascertain the specific effects of TCE. Methodological papers, network meta-analyses, and reevaluations of SRs. Duplicate publications or incomplete SRs.

### 2.3. Literature screening and data extraction

Two independent researchers conducted the literature screening and data extraction per the predefined inclusion and exclusion criteria. In cases of discrepancies, consensus was reached through consultation with a third researcher. The bibliographic records obtained from the searches were imported into the Note Express software to remove duplicates. Full texts were reviewed after an initial screening of the titles and abstracts, with detailed documentation of reasons for exclusion.

Data extraction was carried out using the Microsoft Excel software. The extracted data encompassed the following categories: general information (study title, authors, publication year, corresponding author and affiliation, journal title and impact factor, search period, number of included trials, sample size, registration or publication plan status, mention of Preferred Reporting Items for Systematic Reviews and Meta-Analyses [PRISMA], searched databases, literature quality assessment tools, statistical software, funding support, and the use of the Grading of Recommendations, Assessment, Development, and Evaluation [GRADE] system), study subjects (COPD stage, diagnostic criteria, and age), intervention measures (type of TCE and control group interventions), meta-analysis results, and conclusions.

### 2.4. Calculation of repetition rate

The increasing number of SRs raises concerns about potential overlaps in primary literature due to the replication of SRs on the same topic, which can have implications for data analysis and the assessment of evidence quality.^[19]^ The assessment of overlap involves creating an overlap matrix for SRs and computing the “corrected covered area” (CCA) using the formula: CCA = (*n* − *r*)/(*rc* − *r*); where *n* represents the total number of primary studies included in the SRs, *r* represents the total number of primary studies remaining in the SRs after removing duplicates, and *c* denotes the number of SRs included in the present overview. Notably, 0 to 5 indicates a slight overlap, 6 to 10 suggests a moderate overlap, 11 to 15 implies a high overlap, and > 15 signifies a very high overlap.

### 2.5. Quality assessment of literature

The quality of the SRs was evaluated using the AMSTAR II tool,^[20]^ which comprises 16 items, each with 3 options: “Yes,” “Partial Yes,” and “No.” Among these items, Items 2, 4, 7, 9, 11, 13, and 15 are critical for evaluating the overall quality of the SR. The absence of critical items or the presence of only one non-critical item indicates high quality. The presence of more than one non-critical item indicates moderate quality. The presence of one critical item, with or without non-critical items, indicates low quality. The presence of more than one critical item, with or without non-critical items, indicates very low quality.

### 2.6. Quality grading of evidence

The grading of evidence followed the general principles of the GRADE system.^[21]^ All included SRs were initially regarded as high-level evidence. The quality of evidence was then categorized into 4 levels based on risk of bias, inconsistency, indirectness, imprecision, and publication bias. The 4 categories were designated as high, moderate, low, and very low. The overall quality of evidence for outcome indicators was determined using the lowest evidence level.

### 2.7. Data analysis

Descriptive summaries were provided for the outcome indicators included in the SRs. GRADE evidence levels, combined effect sizes from the included SRs, statistical heterogeneity (*I*^2^) of individual effect sizes, 95% confidence intervals (CIs), number of original studies included, and total sample sizes were presented. Additionally, original study findings were collectively analyzed to assess the results of different therapeutic interventions for the same outcome indicators, considering the conclusions drawn from the original texts.

The conclusions from the original texts were categorized as follows: “Effective”: Both the meta-analysis results and the original text explicitly reported the effectiveness of the intervention. “Potentially Effective”: The meta-analysis results suggested effectiveness, although the original text did not explicitly report the intervention’s effectiveness. “No Difference”: Both the meta-analysis results and the original text indicated no significant differences between the intervention and control measures. “Uncertain”: The meta-analysis results suggested no significant differences, but the original text explicitly reported effectiveness.

## 3. Results

### 3.1. Literature search

**Figure 1.**
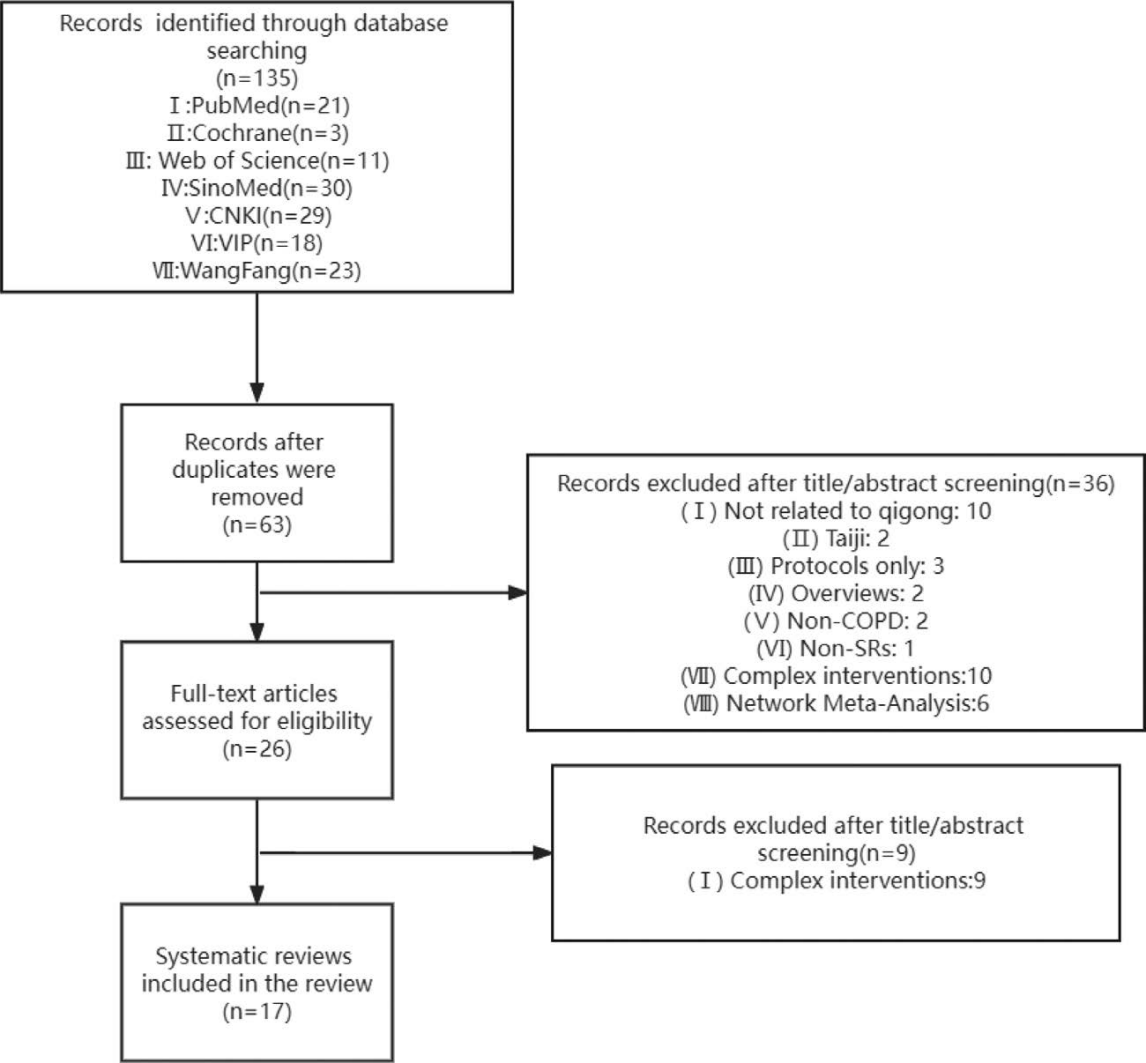
Literature selection process and outcomes.

Our preliminary search yielded 135 citations of SRs published in either English or Chinese as of April 5, 2023. After removing duplicates, 63 unique citations were obtained. Subsequently, a review of titles and abstracts led to the identification of 26 articles. After full-text assessments, 17 SRs (10 in Chinese and 7 in English) focusing on TCE for COPD were included in the final analysis.^[22–38]^ A flowchart depicting the literature selection process and outcomes is presented in Figure 1.

### 3.2. Characteristics of included literature

A total of 17 SRs on TCE for COPD were included in this study. These SRs mainly encompassed the use of Liuzijue (n = 7, 41.1%), Baduanjin (n = 6, 35.3%), Wuqinxi (n = 2, 11.8%), and Health Qigong (n = 2, 11.8%) and were predominantly published by researchers from China (n = 17, 100%) and Korea (n = 1, 5.9%) between 2015 and 2022. The highest number of publications was in 2018 (n = 4, 23.5%). Among the journals publishing these SRs, the “International Journal of Environmental Research and Public Health” (IF_2022_ = 4.614) and the “Journal of Nursing (China)” ranked first, with each publishing 2 articles. “BMC Complementary and Alternative Medicine” (IF_2022_ = 4.782) had the highest impact factor. Each SR searched at least 5 Chinese and English databases, with one SR searching up to 11 databases. PubMed, Cochrane Library (n = 17, 100%), CNKI, and WanFang (n = 17, 100%) were the most commonly used databases. Revman (n = 14, 82.4%) was the most commonly used statistical software for the meta-analyses. None of the SRs used the GRADE system to evaluate the quality of evidence of clinical outcomes. Four SRs (23.5%) explicitly stated adherence to the PRISMA statement for reporting, while 2 SRs (11.8%) were registered on the International Prospective Register of Systematic Reviews (PROSPERO) website. A total of 12 SRs (70.6%) received funding support, with 5 of them being funded by national-level grants.

All 17 SRs exclusively included randomized controlled trials (RCTs). Notably, 9 to 40 RCTs were included per SR. The included SRs involved between 573 and 3137 cases (average = 1336 cases). The number of outcome indicators in the SRs ranged from 4 to 22, with lung function indicators, 6MWD, and quality of life measures (SGRQ and CAT) being the primary focus. Two SRs did not predefine the outcome indicators and only stated: “including at least one health outcome.” Additionally, primary and secondary outcome indicators were not defined in 13 SRs (76.5%). The most common primary outcome indicators were 6MWD (n = 4, 100%), FEV1 (n = 3, 75%), and FEV1% (n = 3, 75%), whereas the most common secondary outcome indicators were SGRQ (n = 3, 75%), CAT (n = 2, 50%), and MRC (n = 2, 50%).

Twelve SRs (76.5%) used the Cochrane Risk of Bias assessment tool to evaluate the quality of the included RCTs. The main issues identified in the primary studies included selection bias (failure to mention allocation concealment), reporting bias (not registered on clinical trial platforms), performance bias (not mentioning blinding of participants), and attrition bias (failure to account for dropouts and not using intention-to-treat analysis). Overall, the assessment results indicated a low quality of the included RCTs. Table S1, Supplemental Digital Content, http://links.lww.com/MD/N36 presents more detailed characteristics of the SRs.

### 3.3. Original study replication rate

**Figure 2.**
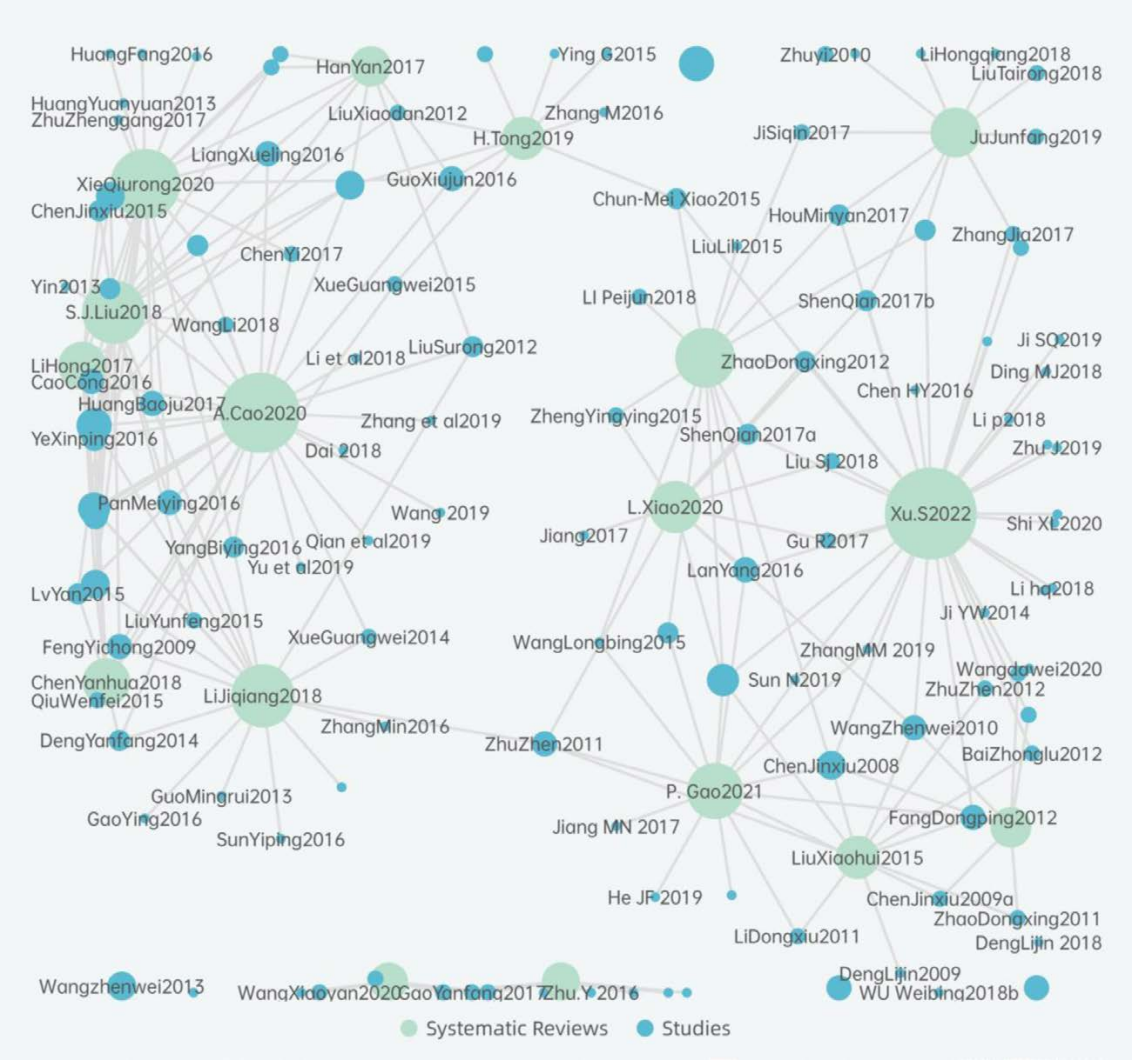
Overlapping node link diagram. In Figure 2. SR is indicated in green bubble, RCT is indicated in blue bubble, a greater number of lines and larger bubbles for original studies represent higher inclusion frequency, while more lines and larger bubbles for SRs indicate a greater number of included original studies. SR = systematic reviews.

**Figure 3.**
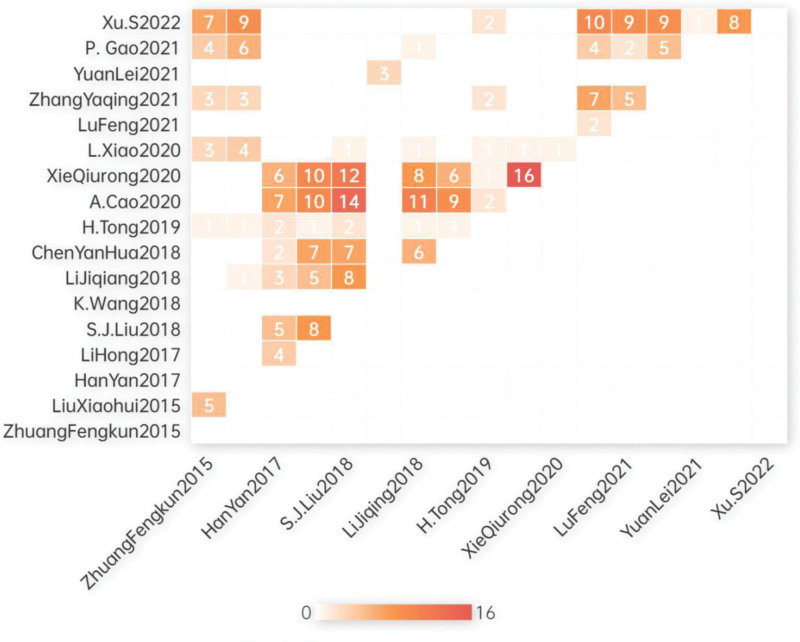
Overlapping heat maps. In Figure 3. darker colors indicate more overlap between the 2 SRs, the numbers in the figure represent the count of original studies that overlapped between the 2 SRs. SR = systematic reviews.

The number of original studies and deduplicated studies were 275 and 124, respectively. The CCA, calculated as CCA = (275 − 166)/(17 × 166 − 166) = 0.041, indicated a slight overlap. Overlap rates were calculated for specific TCE types as follows: Liuzijue (CCA = 0.19), Baduanjin (CCA = 0.28), Wuqinxi (CCA = 0.23), and Health Qigong (CCA = 0.034). The average overlap rate was CCA = 0.18, indicating a slight overlap. Table S2, Supplemental Digital Content, http://links.lww.com/MD/N37 presents the overlap matrix. The network diagram in Figure 2 and the heatmap in Figure 3 were used to visualize the overlaps. In Figure 2, a greater number of lines and larger bubbles for original studies represent higher inclusion frequency, while more lines and larger bubbles for SRs indicate a greater number of included original studies. In Figure 3, darker colors indicate more overlap between the 2 SRs.

### 3.4. AMSTAR 2 Scale Evaluation

**Figure 4.**
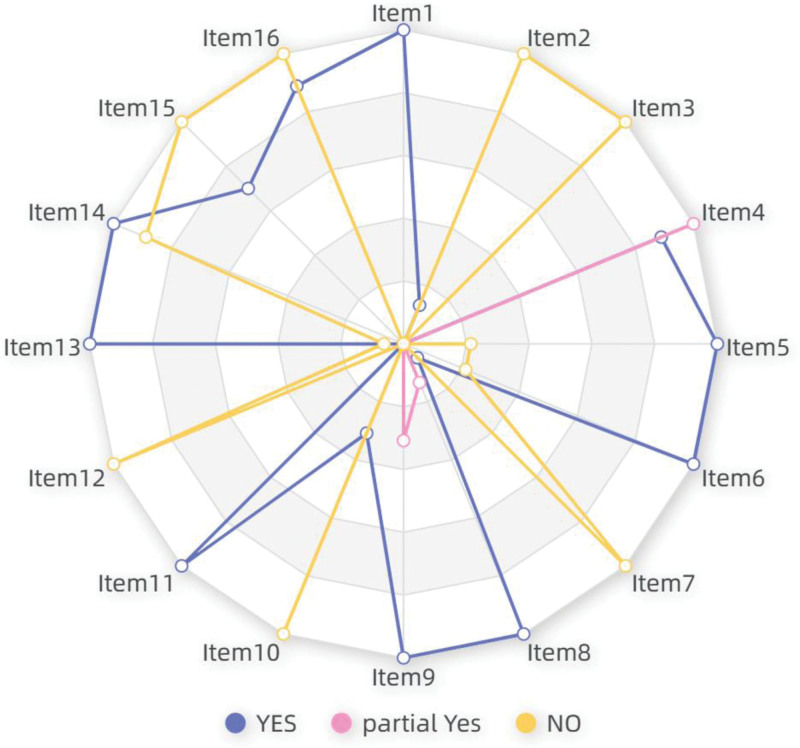
AMSTAR 2 methodologic quality of SR. The quality of the SRs was evaluated using the AMSTAR II tool, which comprises 16 items, each with 3 options: “Yes,” “Partial Yes,” and “No.” Overall, the quality of the included studies was relatively low. SR = systematic reviews.

Only one SR^[37]^ (Liuzijue combined with conventional treatment vs conventional treatment alone) had an overall methodological quality rated as “low,” while the remaining 16 SRs were rated as “very low.” The detailed scoring results are presented in Table S3, Supplemental Digital Content, http://links.lww.com/MD/N38. The radar chart in Figure 4 displays the results of key items. Two SRs (11.8%, 95% CI: 3.29–34.33) formulated a research protocol and registered their study plans on the PROSPERO website. All 17 SRs employed comprehensive literature search strategies (100%, 95% CI: 81.57–100). Additionally, Key Items 8 and 9 were scored “yes” and “partial yes,” respectively, for all included SRs. One SR provided a list of excluded studies and reasons for their exclusion (5.9%, 95% CI: 1.05–26.98). All included SRs employed reasonable methods for assessing the risk of bias in included primary studies (100%, 95% CI: 81.57–100), with 5 SRs (2 studies using only the Jadad scale and 3 studies using only the PEDro scale) rated as “partial yes.” All included SRs used appropriate statistical methods (100%, 95% CI: 81.57–100). One SR did not adequately address bias risk in interpreting/discussing results (94.1%, 95% CI: 73.02–98.95). Seven SRs comprehensively evaluated publication bias and discussed its potential impact on study outcomes (41.2%, 95% CI: 21.61–64).

Furthermore, the majority of included SRs addressed Problem/Patient/Population, Intervention/Indicator, Comparison, and Outcome (PICO) components in the research question and inclusion criteria (100%, 95% CI: 81.57–100); performed study selection in duplicate (82.4%, 95% CI: 58.97–93.81); performed data extraction in duplicate (82.4%, 95% CI: 58.97–93.81); and described the included studies in adequate detail (100%, 95% CI: 81.57–100). Moreover, 35.3% (95% CI: 17.31–58.7) of the included SRs reported on the sources of funding for the included studies. None of the SRs assessed the potential impact of risk of bias in individual studies on the results of the meta-analysis (0%, 95% CI: 0–18.43). A significant proportion (52.9%, 95% CI: 30.96–73.83) of the included SRs adequately investigated publication bias and discussed its likely impact on the study findings. Similarly, a large number of the SRs (52.9%, 95% CI: 30.96–73.83) reported potential sources of conflict of interest, including any funding. Overall, the quality of the included studies was relatively low.

### 3.5. Outcome indicators and evidence quality grading

A total of 28 outcome indicators were included in the literature. However, this study analyzed only 10 of these outcome indicators. The primary outcome indicators analyzed included lung function (FEV1, FEV1%, FVC, and FEV1/FVC%), exercise endurance (6MWD), and quality of life (CAT, MRC/mMRC, and SGRQ). The secondary outcome indicators analyzed included TCM symptom scale and adverse events.

The GRADE system was used to assess the quality of 98 pieces of evidence for the 10 outcome indicators. The quality of evidence ranged from very low to moderate, with no studies classified as high-quality. The results revealed 30 pieces of very low-quality evidence (30.6%), 51 pieces of low-quality evidence (52.0%), and 17 pieces of moderate-quality evidence (17.3%) (Table S4, Supplemental Digital Content, http://links.lww.com/MD/N39). The low grading levels may be attributed to limitations in the original studies (98%), with all original RCTs having serious risks of bias, particularly in randomization, allocation concealment, and blinding methods. The low grading levels may also be attributed to publication bias (82.7%), inconsistency (26.5%), and imprecision (10.2%).

#### 3.5.1. Outcome indicators and conclusions

##### 3.5.1.1. (一) Primary outcome indicators

###### 3.5.1.2.1. FEV1

Fourteen SRs consistently demonstrated that Baduanjin (n = 6), Liuzijue (n = 5), Wuqinxi (n = 2), and Health Qigong (n = 1) significantly improved FEV1 compared to conventional treatment.

The GRADE assessment of evidence quality for FEV1 revealed that the pieces of evidence provided by 3 studies were of very low quality. Seven studies provided low-quality evidence, while 4 studies provided moderate-quality evidence. The overall evidence quality for FEV1, based on intervention type, was determined using the lowest evidence grade. Consequently, the level of evidence for Liuzijue and Health Qigong interventions was low, while that of Baduanjin and Wuqinxi was very low (Table 1).

**Table 1:** Summary table of SR efficacy of TCE in FEV1 outcomes.

Author	Intervention	Effect size	95% CI	heterogeneity	RCTs (n)	Sample	GRADE	Conclusion
S. J. Liu 2018^[26]^	Baduanjin	Hedge’s g = 0.47	0.22, 0.73	*I^2^* = 68%	10	809	Moderate	Effective
K. Wang 2018^[27]^	Wuqinxi	SMD = 0.44	0.12, 0.77	*I^2^* = 33%	3	208	Moderate	Effective
P. Gao 2021^[37]^	Liuzijue	MD = 0.19	0.13, 0.24	*I*^2^ = 5%	7	560	Moderate	Effective
Li Hong 2017^[25]^	Baduanjin	MD = 0.30	0.14, 0.46	*I^2^* = 75%	4	346	Moderate	Potentially Effective
Chen Yanhua 2018^[29]^	Baduanjin	MD = 0.25	0.12, 0.38	*I^2^* = 67%	7	525	Low	Effective
Xie Qiurong 2020^[32]^	Baduanjin	SMD = 1.05	0.56, 1.55	*I^2^* = 94%	12	1201	Low	Effective
L. Xiao 2020^[33]^	Liuzijue	MD = 0.23	0.07, 0.38	*I*^2^ = 83%	8	502	Low	Effective
Xu S 2022^[38]^	Liuzijue	MD = 0.17	0.09, 0.25	*I*^2^ = 68%	13	936	Low	Effective
Zhuang Fengkun 2015^[22]^	Liuzijue	MD = 0.08	-0.04, 0.19	*I^2^* = 51%	4	225	Low	Potentially Effective
Liu Xiaohui 2015^[23]^	Liuzijue	MD = 0.10	0.01, 0.18	*I^2^* = 0%	5	247	Low	Effective
H. Tong 2019^[30]^	Health Qigong	MD = 0.32	0.09, 0.56	*I^2^* = 90%	5	449	Low	Effective
Han Yan 2017^[24]^	Baduanjin	MD = 0.26	0.14, 0.37	*I^2^* = 82%	5	450	Very low	Effective
A. Cao 2020^[31]^	Baduanjin	MD = 0.23	0.15, 0.31	*I^2^* = 83%	17	1395	Very low	Effective
Yuan Lei 2021^[36]^	Wuqinxi	MD = 0.39	0.21, 0.57	*I^2^* = 73%	4	258	Very low	Effective

FEV1 = forced expiratory volume in 1second, RCT = randomized controlled trial, SR = systematic reviews, TCE = traditional Chinese exercise.

###### 3.5.1.2.2. FEV1%

Fifteen SRs consistently demonstrated that Liuzijue (n = 6), Baduanjin (n = 5), Wuqinxi (n = 2), and Health Qigong (n = 2) interventions improved FEV1% compared to conventional treatment.

The GRADE assessment revealed that the pieces of evidence provided by 3 studies of very low quality. Eight studies provided low-quality evidence, while 4 studies provided moderate-quality evidence. For the intervention types, Baduanjin received a low-quality evidence rating, while Liuzijue and Wuqinxi received a very low-quality evidence rating (Table 2).

**Table 2:** Summary table of SR efficacy of TCE in FEV1% outcomes.

Author	Intervention	Effect size	95% CI	Heterogeneity	RCTs (n)	Sample	GRADE	Conclusion
Xie Qiurong 2020^[32]^	Baduanjin	SMD = 0.50	0.24, 0.76	*I^2^* = 86%	15	1848	Moderate	Effective
S. J. Liu 2018^[26]^	Baduanjin	Hedge’s g = 0.38	0.21, 0.56	*I^2^* = 54%	14	1417	Moderate	Effective
K. Wang 2018^[27]^	Wuqinxi	SMD = 0.59	0.24, 0.93	*I^2^* = 63%	6	468	Moderate	Effective
P. Gao 2021^[37]^	Liuzijue	MD = 9.71	8.44, 10.98	*I^2^* = 57%	11	861	Moderate	Effective
Liu XiaoHui 2015^[23]^	Liuzijue	MD = 3.08	0.18, 5.97	*I^2^* = 24%	5	247	Low	Effective
L. Xiao 2020^[33]^	Liuzijue	MD = 7.59	2.92, 12.26	*I*^2^ = 97%	10	580	Low	Effective
Xu S 2022^[38]^	Liuzijue	MD = 6.04	3.43, 8.65	*I*^2^ = 69%	16	904	Low	Effective
Li Hong 2017^[25]^	Baduanjin	MD = 6.86	4.13, 9.60	*I^2^* = 67%	9	985	Low	Potentially effective
Han Yan 2017^[24]^	Baduanjin	MD = 6.02	5.02, 7.01	*I^2^* = 36%	7	848	Low	Effective
Chen Yanhua 2018^[29]^	Baduanjin	MD = 6.71	4.25, 9.18	*I^2^* = 68%	10	1005	Low	Effective
H. Tong 2019^[30]^	Health Qigong	MD = 6.04	2.58, 9.5	*I^2^* = 61%	5	455	Low	Effective
Li Jiqiang 2018^[28]^	Health Qigong	MD = 5.35	2.58, 8.12	*I^2^* = 86%	19	1541	Low	Effective
Yuan Lei 2021^[36]^	Wuqinxi	MD = 8.44	0.40, 16.48	*I^2^* = 95%	4	324	Very low	Effective
Lu Feng 2021^[34]^	Liuzijue	MD = 5.97	2.18, 9.77	*I^2^* = 87%	6	438	Very low	Effective
Zhuang Fengkun 2015^[22]^	Liuzijue	MD = 4.43	1.31, 10.18	*I^2^* = 57%	3	145	Very low	Potentially effective

FEV1 = forced expiratory volume in 1 second, RCT = randomized controlled trial, SR = systematic reviews, TCE = traditional Chinese exercise.

###### 3.5.1.2.3. FVC

Seven SRs consistently demonstrated that both Baduanjin (n = 6) and Liuzijue (n = 7) significantly increased FVC compared to conventional treatment.

The GRADE assessment revealed that the pieces of evidence provided by 6 studies were of low quality, while 1 study provided moderate-quality evidence. Both Baduanjin and Liuzijue interventions received a low-level evidence rating for improvements in FVC and no intervention received a very low-quality rating (Table 3).

**Table 3:** Summary table of SR efficacy of TCE in FVC outcomes.

Author	Intervention	Effect size	95% CI	Heterogeneity	RCTs (n)	Sample	GRADE	Conclusion
S. J. Liu 2018^[26]^	Baduanjin	Hedge’s g = 0.39	0.22, 0.56	*I^2^* = 57%	8	674	Moderate	Effective
Chen Yanhua 2018^[29]^	Baduanjin	MD = 0.16	0.01, 0.31	*I^2^* = 42%	6	423	Low	Effective
A. Cao 2020^[31]^	Baduanjin	MD = 0.19	0.08, 0.30	*I^2^* = 61%	13	1033	Low	Effective
Lihong 2017^[25]^	Baduanjin	MD = 0.34	0.13, 0.54	*I^2^* = 0%	3	244	Low	Potentially effective
Xie Qiurong 2020^[32]^	Baduanjin	SMD = 0.26	0.03, 0.50	*I^2^* = 68%	9	933	Low	Effective
Han Yan 2017^[24]^	Baduanjin	MD = 0.27	0.06, 0.48	*I^2^* = 0%	7	848	Low	Effective
Xu S 2022^[38]^	Liuzijue	MD = 0.02	−0.24, −0.29	*I*^2^ = 61%	4	249	Low	Effective

FVC = forced vital capacity, RCT = randomized controlled trial, SR = systematic reviews, TCE = traditional Chinese exercise.

###### 3.5.1.2.4. FEV1/FVC%

Fourteen SRs consistently demonstrated that both Baduanjin (n = 5), Liuzijue (n = 5), Wuqinxi (n = 2), and Health Qigong (n = 2) significantly improved FEV1/FVC% compared to conventional treatment. One SR reported no significant difference in FEV1/FVC% for adults with stable-phase COPD following Baduanjin intervention (lasting ≥ 3 months) compared to conventional treatment. From the consistency of the conclusions of fifteen SRs, Liuzijue, wuqinxi and Health Qigong showed consistent beneficial effects on the improvement of FEV1/FVC%.

The GRADE assessment for FEV1/FVC% revealed that 7 studies provided very low-quality evidence, while 6 studies provided low-quality. Two studies provided moderate-quality evidence. The overall quality of evidence for Baduanjin, Wuqinxi, and Liuzijue interventions in improving FEV1/FVC% were all very low (Table 4).

**Table 4:** Summary table of SR efficacy of TCE in FEV1/FVC% outcomes.

Author	Intervention	Effect size	95% CI	Heterogeneity	RCTs (n)	Sample	GRADE	Conclusion
S. J. Liu 2018^[26]^	Baduanjin	Hedge’s g = 0.53	0.35, 0.71	*I^2^* = 53%	13	1284	Moderate	Effective
K. Wang 2018^[27]^	Wuqinxi	SMD = 0.65	0.37, 0.93	*I^2^* = 44%	6	418	Moderate	Effective
Li Hong 2017^[25]^	Baduanjin	MD = 4.50	1.84, 7.16	*I^2^* = 73%	8	905	Low	Potentially Effective
Xie Qiurong 2020^[32]^	Baduanjin	SMD = 0.44	0.20, 0.68	*I^2^* = 83%	14	1762	Low	Effective
Li Jiqiang 2018^[28]^	Health Qigong	MD = 2.53	0.38, 4.68	*I^2^* = 86%	18	1461	Low	Effective
H. Tong 2019^[30]^	Health Qigong	MD = 2.66	1.32, 2.26	*I^2^* = 47%	6	535	Low	Effective
L. Xiao 2020^[33]^	Liuzijue	MD = 06.81	3.22, 10.4	*I*^2^ = 95%	12	769	Low	Effective
P. Gao 2021^[37]^	Liuzijue	MD = 4.81,	2.12, 7.51	*I*^2^ = 83%	9	890	Low	Effective
Chen Yanhua 2018^[29]^	Baduanjin	MD = 4.90	19.61, 41.53	*I^2^* = 90%	8	629	Very Low	Effective
A. Cao 2020^[31]^	Baduanjin	MD = 3.85	2.19, 5.51	*I^2^* = 74%	20	1808	Very Low	Effective
Lu Feng 2021^[34]^	Liuzijue	MD = 4.99	0.71, 9.26	*I^2^* = 95%	8	611	Very Low	Effective
Xu S 2022^[38]^	Liuzijue	MD = 6.95	3.06, 10.83	*I*^2^ = 95%	19	1186	Very Low	Effective
Yuan Lei 2021^[36]^	Wuqinxi	MD = 10.39	5.44, 15.35	*I^2^* = 97%	8	577	Very Low	Effective
Zhuang Fengkun 2015^[22]^	Liuzijue	MD = 1.94	1.73, 5.61	*I^2^* = 51%	3	180	Very Low	Potentially Effective
Han Yan 2017^[24]^	Baduanjin	MD = 3.63	−0.18, 7.43	*I^2^* = 33%	6	775	Very Low	Uncertain

FEV1 = forced expiratory volume in 1 second, FVC = forced vital capacity, RCT = randomized controlled trial, SR = systematic reviews, TCE = traditional Chinese exercise.

###### 3.5.1.2.5. 6MWD

All 17 SRs consistently demonstrated that Liuzijue (n = 7), Baduanjin (n = 6), Wuqinxi (n = 2), and Health Qigong (n = 2) significantly improved 6MWD compared to conventional treatment.

The GRADE assessment for 6MWD revealed that 3 studies provided very low-quality evidence, while 10 studies provided low-quality evidence. Three studies provided moderate-quality evidence. The study conducted by Chen et al^[29]^ exhibited significant unexplained heterogeneity, preventing quantitative analysis. Consequently, a qualitative analysis was conducted, making the assessment of evidence quality challenging. The quality of evidence for Health Qigong intervention in improving exercise endurance was of low quality, while those of Liuzijue, Baduanjin, and Wuqinxi were of very low quality (Table 5).

**Table 5:** Summary table of SR efficacy of TCE in 6MWD outcomes.

Author	Intervention	Effect size	95% CI	Heterogeneity	RCTs (n)	Sample	GRADE	Conclusion
Li Hong 2017^[25]^	Baduanjin	MD = 56.35	37.55, 75.16	*I^2^* = 66%	6	476	Moderate	Effective
S. J. Liu 2018^[26]^	Baduanjin	Hedge’s g = 0.69	0.44, 0.94	*I^2^* = 66%	10	886	Moderate	Effective
P. Gao2021^[37]^	Liuzijue	MD = 21.89	14.67, 29.11	*I^2^* = 43%	6	805	Moderate	Effective
Zhuang Fengkun 2015^[22]^	Liuzijue	MD = 24.28	8.73, 39.82	*I^2^* = 30%	4	306	Low	Effective
Liu Xiaohui 2015^[23]^	Liuzijue	MD = 22.62	10.49, 34.75	*I^2^* = 0%	5	326	Low	Effective
L. Xiao 2020^[33]^	Liuzijue	MD = 17.78	7.97, 27.58	*I*^2^ = 0%	6	274	Low	Effective
Zhang Yaqing 2021^[35]^	Liuzijue	MD = 38.47	17.95, 59.00	*I^2^* = 74%	9	475	Low	Effective
Xu S 2022^[38]^	Liuzijue	MD = 33.06	23.73, 42.38	*I*^2^ = 78%	17	1297	Low	Effective
Han Yan 2017^[24]^	Baduanjin	MD = 45.27	40.11, 50.42	*I^2^* = 28%	4	346	Low	Effective
Xie Qiurong 2020^[32]^	Baduanjin	SMD = 1.33	0.97, 1.68	*I^2^* = 83%	12	895	Low	Effective
K. Wang 2018^[27]^	Wuqinxi	SMD = 1.18	0.53, 1.84	*I^2^* = 85%	5	325	Low	Effective
Li Jiqiang 2018^[28]^	Health Qigong	MD = 44.46	20.59, 68.34	*I^2^* = 95%	12	886	Low	Effective
H. Tong 2019^[30]^	Health Qigong	MD = 30.57	19.61, 41.53	*I^2^* = 90%	8	629	Low	Effective
A. Cao 2020^[31]^	Baduanjin	MD = 43.83	29.47, 58.20	*I^2^* = 96%	18	1562	Very low	Effective
Yuan Lei 2021^[36]^	Wuqinxi	MD = 63.42	34.06, 92.79	*I^2^* = 93%	4	278	Very low	Effective
Lu Feng 2021^[34]^	Liuzijue	MD = 39.28	11.98, 66.58	*I^2^* = 74%	4	329	Very low	Effective

6MWD = 6-minute walk distance, RCT = randomized controlled trial, SR = systematic reviews, TCE = traditional Chinese exercise.

###### 3.5.1.2.6. CAT

Nine included SRs demonstrated that Liuzijue (n = 4), Baduanjin (n = 2), and Health Qigong (n = 2) significantly improved CAT scores compared to conventional treatment. One SR (Xie et al^[30]^) observed no significant differences in CAT scores between Baduanjin and control groups, and the impact of Baduanjin on the quality of life of COPD patients was unclear. Both Liuzijue and Health Qigong showed consistent beneficial effects on the improvement of CAT scores.

The GRADE assessment revealed that 4 studies provided very low-quality evidence, while 5 studies provided low-quality evidence. No study provided moderate-quality evidence. Liuzijue intervention for COPD received a low level of evidence rating, while Baduanjin and Health Qigong received a very low level of evidence rating (Table 6).

**Table 6:** Summary table of SR efficacy of TCE in CAT outcomes.

Author	Intervention	Effect size	95% CI	Heterogeneity	RCTs (n)	Sample	GRADE	Conclusion
L. Xiao 2020^[33]^	Liuzijue	MD = −2.29	−3.27, −1.3	*I*^2^ = 56%	4	341	Low	Effective
Lu Feng 2021^[34]^	Liuzijue	MD = −2.50	−2.99, −2.01	*I^2^* = 0%	5	415	Low	Effective
Zhang Yaqing 2021^[35]^	Liuzijue	MD = −2.69	−3.34, −2.03	*I^2^* = 4%	4	266	Low	Effective
Xu S 2022^[38]^	Liuzijue	MD = −2.04	−2.77, −1.30	*I*^2^ = 55%	7	554	Low	Effective
Li Jiqiang 2018^[28]^	Health Qigong	MD = −4.18	−5.52, −2.84	*I^2^* = 6%	4	262	Low	Effective
Chen Yanhua 2018^[29]^	Baduanjin	MD = −1.84	−3.50, −0.19	*I^2^* = 78%	5	679	Very Low	Effective
A. Cao 2020^[31]^	Baduanjin	MD = −2.56	−4.13, −1.00	*I^2^* = 78%	7	802	Very Low	Effective
H. Tong 2019^[30]^	Health Qigong	MD = −5.54	−9.49, −1.59	*I^2^* = 84%	3	258	Very Low	Effective
Xie Qiurong 2020^[32]^	Baduanjin	SMD = −0.56	−1.24, 0.12	*I^2^* = 87%	3	443	Very Low	No Difference

CAT = COPD Assessment Test, RCT = randomized controlled trial, SR = systematic reviews, TCE = traditional Chinese exercise.

###### 3.5.1.2.7. MRC/mMRC

Six SRs demonstrated that Liuzijue significantly improved MRC/mMRC scores compared to control interventions.

The GRADE assessment revealed that 4 studies provided low-quality evidence, while 2 studies provided moderate-quality evidence. The overall quality of evidence for Liuzijue was low (Table 7).

**Table 7:** Summary table of SR efficacy of TCE in MRC/mMRC outcomes.

Author	Intervention	Effect size	95% CI	Heterogeneity	RCTs (n)	Sample	GRADE	Conclusion
P. Gao 2021-mMRC^[37]^	Liuzijue	MD = −0.73	0.96, 0.50	*I*^2^ = 63%	3	459	Moderate	Effective
Xu S 2022-MRC^[38]^	Liuzijue	MD = −0.37	−0.57, −0.18	*I*^2^ = 15%	2	134	Moderate	Effective
Xu S 2022-mMRC^[38]^	Liuzijue	MD = −0.34	−0.48, −0.20	*I*^2^ = 55%	9	974	Low	Effective
L. Xiao 2020-MRC^[33]^	Liuzijue	MD = −0.73	−1.13, −0.33	*I*^2^ = 62%	3	136	Low	Effective
Lu Feng 2021-mMRC^[34]^	Liuzijue	MD = −0.38	−0.54, −0.21	*I^2^* = 59%	3	231	Low	Effective
Zhang Yaqing 2021-mMRC^[35]^	Liuzijue	MD = −0.55	−0.75, −0.36	*I^2^* = 22%	5	228	Low	Effective

MRC/mMRC = Modified Medical Research Council Dyspnea Scale, RCT = randomized controlled trial, SR = systematic reviews, TCE = traditional Chinese exercise.

###### 3.5.1.2.8. SGRQ

Fifteen SRs consistently demonstrated that Baduanjin significantly improved SGRQ scores compared to control interventions.

The GRADE assessment revealed that 10 studies provided very low-quality evidence, while 5 studies provided low-quality evidence. No study provided moderate-quality evidence. Overall, Baduanjin for improvements in SGRQ scores received a low level of evidence rating (Table 8).

**Table 8:** Summary table of SR efficacy of TCE in SGRQ outcomes.

Author	Intervention	Effect size	95% CI	Heterogeneity	RCTs (n)	Sample	GRADE	Conclusion
L. Xiao 2020^[33]^	Liuzijue	MD = −9.85	−13.13, −6.56	*I*^2^ = 63%	5	197	Low	Effective
Lu Feng 2021^[34]^	Liuzijue	MD = −6.86	−11.56, −2.16	*I^2^* = 62%	3	253	Low	Effective
Xu S 2020-总分	Liuzijue	MD = −6.94	−9.20, −4.67	*I*^2^ = 82%	12	938	Low	Effective
Xie Qiurong 2020^[32]^	Baduanjin	SMD = −1.36	−1.74, −0.98	*I*^2^ = 81%	3	762	Low	Effective
A. Cao 2020^[31]^	Baduanjin	MD=−7.71	−10.54, −4.89	*I^2^* = 54%	4	280	Low	Effective
Zhuang Fengkun2015-Symptom^[22]^	Liuzijue	MD = −6.39	−10.94, −1.84	*I^2^* = 0%	2	123	Very low	Effective
Xu S 2022-Activity^[38]^	Liuzijue	MD = −8.43	−12.62, −4.23	*I*^2^ = 93%	10	667	Very low	Effective
Xu S 2022-Symptom^[38]^	Liuzijue	MD = −7.22	−11.29, −3.15	*I*^2^ = 91%	10	667	Very low	Effective
Xu S 2020-Impact	Liuzijue	MD = −5.89	−9.25, −2.53	*I*^2^ = 86%	10	667	Very low	Effective
Liu Xiaohui 2015-Impact^[23]^	Liuzijue	MD = 7.60	2.34, 12.85	*I^2^* = 54%	3	165	Very low	Effective
Zhuang Fengkun 2015-Activity^[22]^	Liuzijue	WMD = −6.95	−16.89, 3.00	*I^2^* = 76%	2	123	Very low	No Difference
Liu Xiaohui 2015-Symptom^[23]^	Liuzijue	MD = 7.89	−1.43, 17.22	*I^2^* = 90%	3	165	Very low	No Difference
Liu Xiaohui 2015-Activity^[23]^	Liuzijue	MD = 8.96	4.09, 13.83	*I^2^* = 61%	3	165	Very low	No Difference
Zhuang Fengkun 2015-Impact^[22]^	Liuzijue	MD = 0.58	−5.00, 6.16	*I^2^* = 0%	2	123	Very low	No Difference
Zhuang Fengkun 2015-Total^[22]^	Liuzijue	MD = −6.77	−14.76, 1.22	*I^2^* = 66%	2	123	Very low	No Difference

RCT = randomized controlled trial, SGRQ = St. George’s Respiratory Questionnaire, SR = systematic reviews, TCE = traditional Chinese exercise.

##### 3.5.1.2. (二)Secondary outcome measures

###### 3.5.1.2.1. TCM symptom scale

One included SR demonstrated that Liuzijue significantly outperformed the control treatment in improving the TCM Symptom Scale. Furthermore, Liuzijue intervention for COPD received a moderate-level evidence rating (Table 9).

**Table 9:** Summary table of SR efficacy of TCE in TCM Symptom Scale outcomes.

Author	Intervention	Effect size	95% CI	Heterogeneity	RCTs (n)	Sample	GRADE	Conclusion
Xu S 2022^[38]^	Liuzijue	MD = −1.85	−2.86, −0.85	*I*^2^ = 94%	7	529	Moderate	Effective

RCT = randomized controlled trial, SR = systematic reviews, TCE = traditional Chinese exercise, TCM = traditional Chinese medicine.

###### 3.5.1.2.2. Safety assessment

Five SRs reported no TCE-related adverse reactions, while one SR mentioned that only some RCTs addressed safety issues related to Baduanjin, with no adverse reactions reported. The research team reviewed 124 RCTs included in the 17 SRs and found no reports of TCE-related adverse reactions.

### 3.6. Evidence map

The main COPD outcome measures, GRADE evidence levels, and summaries of therapeutic efficacy measures are presented in Table 10. Very low evidence is indicated in red, low evidence is indicated in blue, moderate evidence is indicated in yellow, and high evidence is indicated in green. Additionally, the numbers in the table represent the count of intervention-control comparisons for each outcome.

**Table 10:**
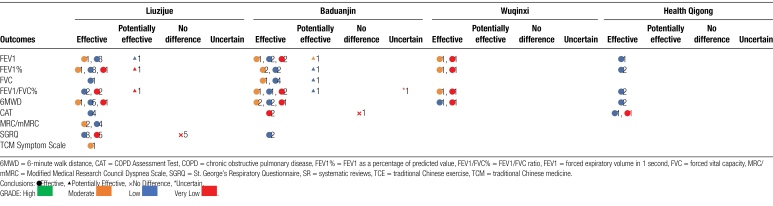
Evidence map of TCE for COPD SR.

## 4. Discussion

### 4.1. Effect of different TCEs in COPD

A comprehensive analysis of the effects of various TCEs on patients with COPD was conducted. Liuzijue (n = 7) significantly improved FEV1, FEV1%, FEV1/FVC%, FVC, 6MWD, MRC/mMRC, and TCM symptom scores. Similarly, Baduanjin (n = 6) significantly improved FEV1, FEV1%, FVC, 6MWD, and SGRQ. Wuqinxi (n = 2) also improved FEV1, FEV1%, FEV1/FVC%, and 6MWD. Furthermore, Health Qigong (n = 2) significantly improved FEV1, FEV1%, FEV1/FVC%, and 6MWD.

The GRADE assessment suggests that Liuzijue (moderate quality) is more effective than conventional treatment in improving TCM symptom scores. Additionally, Liuzijue (low quality) is more effective than conventional treatment in improving FEV1, FVC, CAT, and MRC/mMRC scores. Baduanjin (low quality) demonstrates greater efficacy in improving FEV1%, FVC, and SGRQ scores compared to conventional treatment. Health Qigong (low quality) improves exercise endurance (6MWD) compared to conventional treatment. However, caution should be exercised when interpreting these results. The effectiveness of Baduanjin in improving FEV1/FVC% remains uncertain due to very low-quality evidence. Similarly, the effectiveness of Baduanjin and Liuzijue in improving CAT and SGRQ scores, respectively, were not significantly different compared to control interventions.

In conclusion, the meta-analysis demonstrates that TCEs can improve respiratory function and quality of life among patients with COPD. Liuzijue and Baduanjin emerged as particularly promising interventions, but the quality of evidence varies across different outcome measures. Further high-quality research is required to validate these findings and provide more robust evidence for the effectiveness of TCE in COPD management.

The improvements in the outcome measures may be because TCE enhances the body’s immune function and respiratory muscle strength. The onset of COPD is associated with aberrant inflammatory responses.^[39]^ Modern research has indicated that Liuzijue^[16,40–43]^ potentially decreases serum proinflammatory factors (interleukin 8 [IL-8], tumor necrosis factor [TNF]-α); increases serum fibronectin (Fn) levels; regulates the percentages of peripheral blood T lymphocyte subsets CD3+, CD4+, CD8+, and CD4+/CD8+; modulates the expression of immune factor Notch1 mRNA; mitigates inflammatory responses; boosts immune competence; promotes lung function; and concurrently retards the progressive decline in lung function among stable-phase COPD patients by reducing airway resistance and alleviating respiratory muscle fatigue. These beneficial effects collectively facilitate the rehabilitation of patients with COPD. When combined with medication, Health Qigong^[14]^ markedly reduces the expression of cytokines such as TNF-α and IL-8, leading to a significant enhancement in immune capacity. Baduanjin^[15]^ has been demonstrated to reduce the levels of IL-8 and C-reactive protein in phlegm supernatant, thereby diminishing inflammatory reactions in patients with COPD.

### 4.2. Selection of COPD outcome measures

The selection of COPD outcome measures is closely related to the accuracy of traditional efficacy assessment methods. Analysis of the 17 included SRs revealed the following: Ambiguity in Outcome Measure Definitions: The inclusion criteria were not clear on the definitions of outcome measures. For instance, in the study by Zhuang et al, one of the outcome measures was lung function, yet specific biochemical markers for lung function were not clearly stated. Additionally, the SGRQ scoring scale encompasses 4 components: respiratory symptoms, activity limitation, impact of disease, and total score. However, certain SRs^[31–34]^ failed to specify which component of the SGRQ scale was employed for scoring. This inconsistency between anticipated outcome measures and reported indicators introduces a bias risk, potentially impacting the researchers’ assessment of the reliability of study conclusions. Lack of Primary-Secondary Outcome Distinction: Thirteen SRs (76.5%) did not distinguish between primary and secondary outcome measures. This absence of distinction between primary and secondary indicators could directly contribute to confusion in result interpretation, hampering the attainment of research objectives.^[44]^ Limited Incorporation of TCM Characteristic Measures: We observed that a limited number of SRs incorporated TCM characteristic indicators. Specifically, one SR integrated a TCM syndrome differentiation scale. Syndrome differentiation constitutes a significant outcome measure in the clinical efficacy evaluation of TCM.^[45]^ Lack of safety reporting: Eleven SRs (64.7%) did not report safety issues. The research team found no reports of adverse reactions after reviewing 124 RCTs included in the 17 SRs. It was also noted that some original studies did not report adverse reactions and safety indicators, raising concerns about the clinical implementation of the interventions. Therefore, future studies need to include safety indicators and adverse reactions in the outcome measures of both original and secondary studies. This comprehensive approach assesses the effects and adverse reactions of TCE and provides more robust evidence for clinical practice.

These findings indicate that the accurate selection and definition of COPD outcome measures play a pivotal role in determining the reliability and validity of study conclusions. Furthermore, establishing a clearer distinction between primary and secondary indicators can enhance the interpretation of the results, ultimately contributing to the achievement of the research objectives. Additionally, the incorporation of TCM-specific indicators warrants greater attention, as these factors bear substantial significance within the context of holistic medical evaluation.

### 4.3. Methodological quality of included SRs

Although current evidence suggests that TCE have beneficial effects in cases of COPD, high-quality studies are needed to draw definitive conclusions. Research indicates that neglecting clinical study registration might be attributed to a lack of knowledge and awareness regarding research planning and registration.^[46]^ As of April 2023, 3 related protocols have been made publicly available. Of these, 2 were published in 2018 and 2019.^[47,48]^ To date, there have been no updates or publications of completed SRs in reputable journals. It is also worth noting that if an SR involves an assessment of adverse reactions, it is important to consider including cohort studies.^[49]^

The presence of high-risk bias in the included RCTs could potentially affect the conclusions of the SRs negatively. The primary limitations of the original studies mainly stem from the inadequate implementation of randomization, allocation concealment, and blinding, which are all factors that significantly contributed to the low-GRADE ratings. Given the nature of TCE interventions, it is challenging to blind both patients and therapists during the intervention period. Additionally, standardizing the training criteria among different therapists can be difficult. It is recommended that researchers should not participate in executing a trial, have no direct interaction with the participants, ensure blinding of outcome assessors, and when blinding is infeasible, provide reasons for its non-implementation to reflect the consideration of the importance of blinding. Notably, none of the 17 included SRs used the GRADE system to evaluate the evidence for outcome measures. Research indicates that only 29% of Chinese researchers are aware of the GRADE system,^[50]^ and substantial barriers to comprehending the GRADE system persist among the majority of Chinese researchers.

In summary, while the existing evidence hints at the potential advantages of TCE for patients with COPD, it is imperative to address the aforementioned issues through rigorous research design, SR registration, clarification of inclusion criteria, consideration of bias risks, and the application of appropriate evidence grading tools.

### 4.4. Problems and limitations

1. Following the principles of evidence-based medicine, this study employed the AMSTAR II checklist and GRADE tool to assess the methodological quality and level of evidence in 17 SRs. It is recommended that future studies consider incorporating the ROBIS checklist^[51]^ and adhere to the PRISMA statement.^[52]^ These additional tools will help evaluate the risk of bias in the SRs and enhance reporting standards.
2. This study employed qualitative descriptions to compare the variations in outcomes among different TCE interventions. Future studies could consider employing network meta-analysis to explore efficacy disparities and advantages when treating COPD with different TCE types. Such analyses would serve as valuable references for clinical applications.
3. This study did not identify any Cochrane Systematic Reviews (CSRs) related to TCE interventions for COPD. CSRs are widely regarded as the gold standard of evidence,^[53]^ with high-quality SRs relying on robust evidence from RCTs. Consequently, in-depth exploration remains imperative for evidence-based research into TCE interventions for COPD in the future.

## 5. Future research

1. Given their safety and efficacy, TCE interventions have the potential to alleviate the economic and societal burdens associated with COPD. However, the cost-effectiveness of TCE compared to other interventions for pulmonary rehabilitation remains uncertain. Future research should conduct a thorough health economic analysis of TCE, considering factors such as training costs. This analysis should adopt a “cost-effectiveness” perspective to offer clinical decision-makers a basis for rational resource allocation.
2. Studies on TCE for managing COPD have been centered around stable-phase COPD patients. However, COPD is characterized by a gradual progression and an absence of symptoms in early-stage patients. Additionally, there is a limited awareness of COPD prevention and management approaches in the general population^[54]^. Thus, early intervention using TCE could hold significant implications for reducing the incidence of COPD, but such clinical research remains notably rare.
3. Previous studies^[29]^ have indicated that the effectiveness of TCE interventions for COPD improves with longer durations of intervention. To ascertain the long-term clinical effectiveness of TCE interventions for COPD, future RCTs could be designed with extended follow-up periods. This approach would facilitate the determination of the long-term benefits of TCE in managing COPD.
4. The current outcome measures employed in studies focusing on TCE for COPD have some limitations that could potentially compromise the scientific rigor and practicality of such studies. To address this issue, future studies need to focus on establishing a comprehensive set of core outcome measures specifically tailored to assess the clinical efficacy of TCE in COPD management. This initiative would contribute to standardizing the evaluation of TCE interventions for COPD and provide valuable insights for the assessment of their therapeutic effects.

## Author contributions

**Conceptualization:** Yin Zhu, Ying Lu, Jie Li.

**Data curation:** Lu Han, Jing Wang, Chaoqun Liu, Chaoyang Chen.

**Formal analysis:** Lu Han, Jing Wang, Chaoqun Liu, Chaoyang Chen.

**Investigation:** Lu Han, Jing Wang.

**Methodology:** Lu Han, Jing Wang.

**Project administration:** Yin Zhu.

**Supervision:** Jie Li.

**Validation:** Yin Zhu, Ying Lu, Jie Li.

**Visualization:** Lu Han, Jing Wang.

**Writing – original draft:** Lu Han, Jing Wang.

**Writing – review & editing:** Yin Zhu, Ying Lu, Jie Li.

## Supplementary Material








