# Supplementary material for: Traditional Chinese exercise in chronic obstructive pulmonary disease: An overview of systematic reviews
Source: Medicine (Baltimore). 2024 Jun 28;103(26):e38700. doi: 10.1097/MD.0000000000038700 (PMC11466204; doi:10.1097/MD.0000000000038700)
Supplement: Supplementary file 1 [file medi-103-e38700-s001.docx]

| Supplementary Table 1-1 Basic characteristics of SR literature on TCE in COPD | | | | | | | | | |
| --- | --- | --- | --- | --- | --- | --- | --- | --- | --- |
| No | Autor | Country^*^ | Journals | IF | Search time | Type of trails | Number | Simple | Registration |
| 1 | ZhuangFengkun2015 | China Nanjing | Doctoral dissertation | / | 2014.10 | *RCTs* | 9 | 594 | NR |
| 2 | LiuXiaohui2015 | China Tianjin | Chinese Journal of Practical Nursing (China) | Chinese core journals | 2014.5 | *RCTs* | 10 | 573 | NR |
| 3 | HanYan2017 | China Shanxi | Journal of Nursing (China) | Chinese core journals | 2010.1-2016.12 | *RCTs* | 9 | 960 | NR |
| 4 | Lihong2017 | China Tianjin | Journal of Nursing (China) | Chinese core journals | 2016.9.30 | *RCTs* | 12 | 1177 | NR |
| 5 | S.J.Liu2018 | China HongKong | *Int. J. Environ. Res. Public Health* | 4.614 | 2018.5 | *RCTs* | 20 | 1975 | NR |
| 6 | K.Wang2018 | Korean；China HongKong/Shanghai | *Int. J. Environ. Res. Public Health* | 4.614 | 2018.11 | *RCTs* | 8 | 687 | NR |
| 7 | LiJiqiang2018 | China Guangzhou | Journal of Liaoning University of Chinese Medicine (China) | / | 2018.1.31 | *RCTs* | 20 | 1664 | NR |
| 8 | ChenYanhua2018 | China Chengdu | Chinese Journal of Rehabilitation Medicine (China) | Chinese core journals | 2016.8 | *RCTs* | 12 | 1245 | NR |
| 9 | H.Tong2019 | ChinaBeijing | *BMC Complementary and Alternative Medicine* | 4.782 | 2017.10.8 | *RCTs* | 10 | 993 | NR |
| 10 | A.Cao2020 | China Nanjing | *Clinical Rehabilitation* | 2.884 | 2020.3 | *RCTs* | 31 | 3045 | NR |
| 11 | XieQiurong2020 | China Fujian | Fujian Traditional Chinese Medicine (China) | / | 2013.1-2018.1 | *RCTs* | 25 | 2058 | NR |
| 12 | L.Xiao2020 | China Shanghai | *BMC Complementary Medicine and Therapies* | 2.838 | 2018.8 | *RCTs* | 14 | 920 | *PROSPERO* |
| 13 | LuFeng2021 | China Fujian | TCM research (China) | / | 2019.12.21 | *RCTs* | 13 | 1030 | NR |
| 14 | ZhangYaqing2021 | China Henan | Clinical Research in Traditional Chinese Medicine (China) | / | 2009.7-2019.7 | *RCTs* | 18 | 1036 | NR |
| 15 | YuanLei2021 | China Chengdu | Evidence-based Care (China) | / | 2020.11.11 | *RCTs* | 8 | 578 | NR |
| 16 | P. Gao2021 | China Hunan | *Medicine* | 1.817 | 1970-2020.9 | *RCTs* | 16 | 1039 | *PROSPERO* |
| 17 | Xu.S2022 | China Nanjing | *Complementary Therapies in Medicine* | 3.335 | 2020.8.8 | *RCTs* | 40 | 3137 | NR |

| Supplementary Table 1-2 Basic characteristics of SR literature on TCE in COPD | | | | | | | | | |
| --- | --- | --- | --- | --- | --- | --- | --- | --- | --- |
| Autor | Patients | Intervention | Control | *PRISMA* | Multiple Databases | Quality evaluation | Statistical software | Fund | Grade |
| zhuangFengkun2015 | Stable COPD | Lizijue or Lizijue＋Usual treatment | Notreatment/  Usualtreatment | NR | *PubMed、Cochrane Central Register of Controlled Trials (CENTRAL)*、*EMbase、SinoMed、CNKI*、*WangFang* | *Cochrane*＋*Jadad* | *RevMan5.1* | NR | NR |
| LiuXiaohui2015 | Stable COPD | Lizijue or Lizijue＋control | Non-Lizijue | NR | *PubMed、Cochrane Library、ELSEVIER、CNKI、VIP、WangFang* | *Cochrane* | *RevMan5.3* | NR | NR |
| HanYan2017 | Stable COPD | Baduanjin＋control | Usual treatment | NR | *PubMed、Cochrane Library、Springer、CBM、CNKI、VIP、WangFang、* | *Cochrane* | *RevMan5.0* | Provincial and ministerial | NR |
| Lihong2017 | Stable COPD | Baduanjin＋control | Health guidance or routine medication or other exercise interventions | NR | *PubMed、Cochrane Library、EMbase、Web of Science、CBM、CNKI、WanFang* | *Cochrane* | *RevMan5.3* | NR | NR |
| S.J.Liu2018 | COPD | Baduanjin or Baduanjin＋other | Other interventions | *PRISMA* | *PubMed、Cochrane Library、Web of Science、Scopus、CNKI、Wanfang* | *PEDro* | *Bio. Stat. Inc.* | Provincial and ministerial | NR |
| K.Wang2018 | COPD | Wuqinx | No or  Other interventions | *PRISMA* | *PubMed、Web of Science、 Scopus、CNKI、Wanfang* | *PEDro* | *Bio. Stat. Inc.* | Provincial and ministerial | NR |
| LiJiqiang2018 | Stable COPD | Health Qigong＋  Usual treatment | Usual treatment，including conventional medicine, health guidance or no intervention | NR | *PubMed、Cochrane Library、Embase、CBM、CNKI、VIP、WanFang* | *Cochrane* | *RevMan5.3* | Provincial and ministerial | NR |
| ChenYanhua2018 | Stable COPD | Baduanjin＋Usual care | Other interventions or  Usual care | NR | *PubMed、Cochrane Library、Web of Science、Ovid、EBSCO、Elsevier、Embase、CBM、CNKI、VIP、WanFang* | *Cochrane* | *RevMan5.1* | NR | NR |
| H.Tong2019 | Stable COPD | Health Qigong | conventional medicine or health guidance | NR | *PubMed、Cochrane Library、Web of Science、EMBASE、CNKI、VIP、WanFang* | *Cochrane* | NR | school | NR |
| A.Cao2020 | COPD | Baduanjin＋other interventions | Other interventions，including medication, routine activities, or breathing training | *PRISMA* | *PubMed、Cochrane Library、EMBASE、CBM、CNKI、WanFang* | *Cochrane* | *RevMan5.3* | Department | NR |
| XieQiurong2020 | COPD | BaduanjinorBaduanjin＋Usual treatment | Baduanjin or Baduanjin＋Usual treatment | NR | *PubMed、PEDro、CNKI、VIP、WanFang* | *PEDro* | *RevMan5.3* | National | NR |
| L.Xiao2020 | Stable COPD | Lizijue or simplified Lizijue | conventional medicine or health guidance | NR | *PubMed、Cochrane Library、EMBASE、Web of Science、CNKI、WanFang* | *Cochrane* | *RevMan5.3.5* | National | NR |
| LuFeng2021 | Stable COPD | Lizijueor＋  acupressure, medication, etc | Basic treatment or usual care | NR | *PubMed、Cochrane Library、Web of Science、Embase、CBM、CNKI、VIP、WanFang* | *Jadad* | *RevMan5.2* | Department | NR |
| ZhangYaqing2021 | Stable COPD | Lizijue＋Usual treatment | Usual treatment | NR | *PubMed、Cochrane Library、CBM、CNKI、VIP、WanFang* | *Cochrane* | *RevMan5.3* | National | NR |
| YuanLei2021 | COPD | Wuqinxi | Blank control or conventional control | NR | *PubMed、Cochrane Library、Web of Science、CBM、CNKI、VIP、WanFang* | *Cochrane* | *RevMan5.4* | NR | NR |
| P. Gao2021 | Stable COPD | Lizijue＋Usual treatment | Usual treatment | NR | *PubMed、Cochrane Library、Web of Science、CNKI、WanFang* | *Cochrane* | *RevMan5.3 and Stata14.0* | National | NR |
| Xu.S2022 | COPD | Lizijueor＋Usual treatment | Usual treatment or other exercise | *PRISMA* | *PubMed、Cochrane Library、Embase、CNKI、VIP、WanFang* | *Jadad* | *RevMan5.2* | National | NR |

| Supplementary Table 1-3 Basic characteristics of SR literature on TCE in COPD | | | | | | |
| --- | --- | --- | --- | --- | --- | --- |
| Author | Primary outcome measures | Secondary outcomes | Meta-analysis results (valid outcome measure) | Results of meta-analyses (null and void outcomes) | conclusion | safety |
| zhuangFengkun2015 | ①6MWD  ②Lung function | ①Quality of life | Lizijue vs control：  ①6MWD[MD=24.28,(8.73,39.82),*P*=0.002,*I^2^*=30%],4 trials,306  ②FEV1[MD=0.08,(-0.04,0.19),*P*=0.21,*I^2^*= 51%]],4 trials,225  ③FEV1%[MD=4.43,(1.31,10.18),*P*=0.13,*I^2^*= 57%],3 trials,145  ④FEVl/FVC[MD=1.94,(1.73,5.61),*P*=0.30,*I^2^*= 51%],3 trials,180  ⑤SGRQ-symptoms  [MD=-6.39,(-10.94,-1.84),*P*=0.006,*I^2^*= 0%],2 trials,123 | Lizijue vs control：  ①SGRQ-activity  [WMD=-6.95,-16.89,3.00,*P*=0.17,*I^2^*= 76%],2 trials,123  ②SGRQ-impact  [MD=0.58,-5.00,6.16,*P*=0.84,*I^2^*= 0%],2trials,123  ③SGRQ-total  [MD=-6.77,(-14.76,1.22),*P*=0.002,*I^2^*= 66%],2 trials,123 | Lizijue helps improve exercise tolerance in COPD patients and effectively improves respiratory symptoms in COPD patients  From the other three lung function indicators, Lizijue did not significantly improve the lung function of patients. | NR |
| LiuXiaohui2015 | 6MWD、FEV1、FEV1%、SGRQ | | ①6MWD[MD=22.62,(10.49,34.75),*P*＜0.05,*I^2^*= 0%],5 trials,326  ②FEV1[MD=0.10,(0.01,0.18),*P*＜0.05,*I^2^*=0%],5 trials,247  ③FEV1%[MD=3.08,(0.18,5.97),*P*=0.04,*I^2^*= 24%],5 trials,247  ④SGRQ-impact[MD=7.60,(2.34,12.85),*P*＜0.05,*I^2^*= 54%],3 trials,165 | Lizijue vs control：  ①SGRQ-symptoms  [MD=7.89,(-1.43,17.22),*P*＞0.05,*I^2^*= 90%],3 trials,165  ②SGRQ-activity  [MD=8.96,(4.09,13.83),*P*＜0.05,*I^2^*= 61%],3 trials,165 | Lizijue can improve exercise tolerance, respiratory function, and quality of life in patients with stable COPD, which has important guiding significance for the treatment of stable COPD, and more large-sample, high-quality, multicenter RCTs are needed to verify. | NR |
| HanYan2017 | FEV1、FEV1%、FVC、FEV1/FVC%、6MWD | | Baduanjin vs control：  ①6MWD[MD=45.27,(40.11,50.42),*P*<0.01,*I^2^*= 28%],4 trials,346  ②FEV1[MD=0.26,(0.14,0.37),*P*<0.01,*I^2^*= 82%],5 trials,450  ③FEV1%[MD=6.02,(5.02,7.01),*P*<0.01,*I^2^*= 36%],7 trials,848  ④FVC[MD=0.27,[0.06,0.48),*P*=0.01],3 trials,266 | Baduanjin vs control：  FEV1/FVC%[MD=3.63,(-0.18,7.43),*P*=0.06],6 trials,775 | Baduanjin can improve lung function and exercise tolerance in patients with stable COPD. | NR |
| Lihong2017 | FEV1、FEV1%、FVC、FEV1/FVC%、6MWD | | Baduanjin vs control：  ①6MWD[MD=56.35,(37.55,75.16),*P*＜0.01,*I^2^* = 66%]，6 trials,476  ②FEV1[MD=0.30,(0.14,0.46),*P*＜0.01,*I^2^* =75%],4 trials,346  ③FEV1%[MD=6.86,(4.13,9.60),*P*＜0.01,*I^2^* = 67%],9 trials,985  ④FVC[MD=0.34,(0.13,0.54),*P*＜0.01,*I^2^* =0%],3 trials,244  ⑤FEV1/FVC%[MD=4.50,(1.84,7.16),*P*＜0.01),*I^2^* = 73%],8 trials,905 |  | Baduanjin long-term intervention (>3 months) is more effective in improving lung function and exercise endurance in patients with stable COPD; Although short-term (≤ 3 months) Baduanjin interventions may improve exercise tolerance, the effect of improving lung function is uncertain and further confirmation is needed. | Only a few RCTs mention the safety of Baduanjin exercise. |
| S.J.Liu2018 | at least one symptom or health outcome measure | | Baduanjin vs control：  ①6MWD[Hedge’s g = 0.69,(0.44,0.94),*P*< 0.001,*I^2^* = 66%],10 trials,  ②FEV1[Hedge’s g = 0.47, (0.22,0.73),*P*< 0.001,*I^2^*= 68.01%],10 trials,809  ③FEV1%[Hedge’s g=0.38,(0.21,0.56),*P*<0.001, *I^2^*= 54.74%],14 trials,1417  ④FVC[Hedge’s g=0.39,(0.22,0.56),*P*< 0.001,*I^2^*= 57%],8 trials,674  ⑤FEV1/FVC%[Hedge’s g=0.53,(0.35,0.71),*P*< 0.001,*I^2^*=53.49%],13 trials,1284 |  | Baduanjin as an adjunct therapy may improve exercise capacity and lung function, as well as quality of life, in patients with COPD. | No adverse events were reported |
| K.Wang2018 | at least one symptom or health outcome measure | | Wuqinxi vs control：  ①6-MWT [SMD=1.18,(0.53,1.84),*P*<0.001,*I^2^*= 84.97%],5 trials,  ②FEV1 [SMD=0.44,(0.12,0.77),*P*<0.001,*I^2^*=33.77%],3trials,208  ③FEV1%[SMD=0.59,(0.24,0.93),*P*<0.001,*I^2^*=63.79%],6 trials  ④FEV1/FVC[SMD=0.65,(0.37,0.93),*P*=0.006,*I^2^*=44.32%],6 trials  ⑤CCQ [SMD=1.23,(0.31,2.14),*P*=0.01,*I^2^*=3.32%],3 trials |  | Wuqinxi may have a rehabilitative effect on COPD patients. | No adverse events were reported |
| LiJiqiang2018 | FEV1%、FVC、FEV_1_/FVC%、CAT评分、6MWD | | Health Qigong vs control :  ①6-MWT: 3 months MD=22.10,(12.43,31.78),*P*<0.00001,*I^2^* = 45%]、  6 months[MD=44.46,(20.59，68.34),*P*=0.0003,*I^2^* = 95%],12 trials,885  ②FEV1%:3 months [MD=5.34,(2.70,7.98),*P*<0.0001]、  6 months [MD=5.35,(2.58,8.12),*P*=0.0001],19 trials,1541  ③FEV1/FVC:3months[MD=4.49(1.66,7.31),*P*=0.002,*I^2^* = 36%],  6 months[MD=2.53,(0.38,4.68),*P*=0.02,*I^2^* = 86%],18 trials,1461  ④CAT[MD=-4.18,(-5.52,-2.84),*P*<0.00001,*I^2^* = 6%],4 trials,262 |  | Health Qigong combined with basic care may improve exercise tolerance, lung function, and quality of life in patients with stable COPD. | NR |
| ChenYanhua2018 | FEV_1_、FEV_1_%、FVC、FEV_1_/FVC%、6MWD、CAT | | Baduanjin vs control：  ①FEV1[MD=0.25,(0.12,0.38),*P*＜0.001,*I^2^* = 67%],7 trials,525  ②FEV1%[MD=6.71,(4.25,9.18),*P*＜0.001,*I^2^* = 68%],10 trials,1005  ③FVC[MD=0.16,(0.01,0.31),*P*=0.04，*I^2^* = 42%],6 trials,423  ④FEV1/FVC%[MD=4.90,(2.43,7.38),*P*＜0.001,*I^2^* = 71%],9 trials,925  ⑤CAT[MD=-1.84,(-3.50,-0.19),*P*＜0.05,*I^2^* = 78%],5 trials,679  ⑥6-MWD:qualitative analysis |  | Baduanjin exercise improves lung function, exercise endurance and quality of life in people with COPD. | NR |
| H.Tong2019 | 6MWD、FEV_1_、FEV_1_/FVC%、FEV_1_%、MFTE、CAT、SF-36 | | Health Qigong vs control:  ①6MWD[MD=30.57,(19.61,41.53),*P*<0.00001,*I^2^* = 90%],8 trials,629  ②FEV1[MD=0.32,(0.09,0.56),*P*<0.001,*I^2^* = 90%],5 trials,449  ②FEV1%[MD=6.04,(2.58,9.5),*P*=0.006,*I^2^* = 61%], 5 trials,455  ③FEV1/FVC%[MD=2.66,(1.32,2.26),*P*=0.0001,*I^2^* = 47%],6 trials,535  ④MFTE[MD=0.88,(0.78,0.99),*P*<0.00001,*I^2^* = 0%],2 trials,171  ⑤CAT [MD=−5.54,(−9.49,−1.59,*P*=0.006,*I^2^* = 84],3 trials,258  ⑥SF-36 for General Health[MD=5.22(3.65,6.80),*P*<0.00001,*I^2^* = 42%],2 trials,171 | Health Qigong vs control:  SF-36 for Mental Health[MD=−1.21,(−2.75,0.33),*P*=0.12,,*I^2^* = 15%],2 trials,171 | Health Qigong can improve lung function, exercise capacity, and quality of life in patients with stable COPD. | NR |
| A.Cao2020 | ①6MWD  ②FEV_1_  ③FEV_1_%  ④FVC | ①SGRQ  ②CAT | Baduanjin vs control：  ①FEV1[MD= 0.23,(0.15, 0.31), *P*<0.00001,*I^2^* = 83%],17 trials,1395  ③FVC[MD=0.19,(0.08, 0.30), *P*=0.0007,*I^2^* = 61%],13 trials,1033  ④FEV1/FVC%[MD=3.85,(2.19,5.51),*P*<0.00001,*I^2^* = 74%],20 trials,1808  ⑤6MWD:[MD=43.83,(29.47,58.20),*P*<0.00001,*I^2^* = 96%],18 trials,1562  ⑥SGRQ[MD=−7.71,(−10.54,−4.89),*P*<0.00001,*I^2^* = 54%],4 trials,280  ⑦CAT[MD=−2.56,(−4.13,−1.00), *P*=0.001,*I^2^* = 78%],7 trials,802 |  | Baduanjin exercise can improve exercise capacity, lung function, and quality of life in people with COPD. | No adverse events were reported. |
| XieQiurong2020 | FEV_1_、FEV_1_%、FVC、FEV1/FVC%、6MWD、SGRQ、CAT、WHOQOL-BREF | | Baduanjin vs control：  ①FEV1[SMD＝1.05,(0.56,1.55),*I^2^*＝94%,*P*＜0.0001],12 trials,1201  ②FEV1%[SMD＝0.50,(0.24,0.76),*I^2^*＝86%,*P*＝0.0002],15 trails,1848  ③FVC[SMD＝0.26,(0.03,0.50),*I^2^*＝68%,*P*＝0.03],9 trials,933  ④FEV1/FVC%[SMD＝0.44,(0.20,0.68),*I^2^*＝83%,*P*＝0.0004],14 trials,1762  ⑤6MWD[SMD＝1.33,(0.97,1.68),*I^2^*＝83%,*P*＜0.001],12 trials,895  ⑦SGRQ[SMD＝-1.36,(-1.74,-0.98),I2＝81%,P＜0.001],3 trials,762 | Baduanjin vs control：  CAT[SMD＝-0.56,(-1.24,0.12),*I^2^*＝87%,*P*＝0.11],3 trials,443 | Baduanjin exercise can improve exercise tolerance and improve lung function in patients with COPD, but the effect on patients' quality of life is unclear and further research is needed. | NR |
| L.Xiao2020 | MRC/mMRC、MIP、MEP、6MWD、 30s SST、FEV_1_、FEV_1_%、FVC、FEV_1_/FVC%、SGRQ、CAT | | Lizijue vs control：  ①FEV1[MD＝0.23,(0.07,0.38),*I*^2^＝83%,*P*=0.004],8 trials,502  ②FEV1%[MD＝7.59,(2.92,12.26),*I*^2^＝97%,*P*＝0.001],10 trails,580  ③FEV1/FVC%[MD＝06.81,(3.22,10.4),*I*^2^＝95%,*P*＝0.0002],12 trials,769  ④6MWD[MD＝17.78,(7.97,27.58),*I*^2^＝0%,*P*=0.0004],6 trials,274  ⑤CAT[MD＝-2.29,(-3.27,-1.3),*I*^2^＝56%,*P*＜0.00001],4 trials,341  ⑥SGRQ[MD＝-9.85,(-13.13,-6.56),I^2^＝63%,*P*＜0.00001],5 trials,197  ⑦MRC[MD＝-0.73,(-1.13,-0.33),*I*^2^＝62%,*P*=0.0004],3 trials,136 |  | Lizijue exercise improves dyspnea, exercise tolerance, lung function, and quality of life in patients with stable COPD. However, due to the methodological limitations of Lizijue exercise and the placebo effect, further studies are needed to confirm it. | No adverse events were reported. |
| LuFeng2021 | ①6MWD  ②FEV_1_  ③FEV_1_%  ④FVC | ①SGRQ  ②CAT  ③mMRC | Lizijue vs control：  ①FEV1[MD= 0.19,(0.13, 0.24), *P*<.00001,*I*^2^＝5%],7 trials,560  ②FEV1%[MD=9.71, (8.44, 10.98), *P*<.00001],11 trails,861  ③FEV1/FVC%[MD=4.81, (2.12, 7.51), *P*=.0005],9 trials,890  ④6MWD[MD=21.89, (14.67, 29.11), *P*<.00001],6 trials,805  ⑤CAT and SGRQ[SMD=-0.70, (-1.01, -0.38), *P*<.001],7 trials,780  ⑥mMRC [MD=0.73, ( 0.96, 0.50), *P*<.00001],3 trials,459 |  | Lizijue improved lung ventilation, exercise tolerance, and health-related quality of life in patients with stable COPD. | NR |
| ZhangYaqing2021 | MRC/mMRC、SGRQ、CAT、6MWD、FEV_1_%、FEV_1_/FVC% | | Lizijue vs control：  ①SGRQ[MD=-6.86,(-11.56,-2.16),*P*=0.004,*I^2^* = 62%],3 trials,253  ②CAT[MD=-2.50,(-2.99,-2.01),*P*<0.000 01,*I^2^* = 0%],5 trials,415  ③mMRC[MD=-0.38,(-0.54,-0.21) ,*P*<0.00001,*I^2^* = 59%],3 trials,231  ④6MWD[MD=39.28,(11.98,66.58),*P*=0.005,*I^2^* = 74%],4 trials.329  ⑤FEV1%[MD=5.97,(2.18,9.77),*P*=0.002,*I^2^* =87%],6 trials,438  ⑥FEVl／FVC[MD=4.99,(0.71,9.26),*P*=0.02,*I^2^* = 95%],8 trials,611 |  | Lizijue respiratory exercise in the treatment of patients with stable COPD has obvious rehabilitation effect and accurate clinical effect, which can improve the patient's cough, sputum production, wheezing, chest tightness, shortness of breath and other symptoms, improve the patient's lung ventilation function, improve the patient's exercise endurance and quality of life, and reduce the number of acute exacerbations in patients. | NR |
| YuanLei2021 | ①6MWD | ①SGRQ  ②mMRC  ③FEV_1_  ④FEV_1_% | Lizijue vs control :  ①6MWD:3 months [MD=24.86,(1.98,47.74)、  More 3 months[MD=38.47(17.95,59.00)],*I^2^* = 74%,9 trials,475  ②mMRC[MD=-0.55(-0.75,-0.36),*P*<0.00001,*I^2^* = 22%],5 trials,228  ③CAT[MD=-2.69(-3.34,-2.03),*P*<0.000 01,*I^2^* = 4%],4 trials,266  ④FEV1[MD=0.19(0.06,0.31),*P*=0.01,*I^2^* = 67%],6 trials,337  ⑤FEV1%[MD=6.08(2.55,9.62),*P*=0.0007,*I^2^* = 89%],13 trials,644 |  | Lizijue can improve exercise endurance in patients with chronic obstructive pulmonary disease, reduce dyspnea symptoms, improve quality of life, delay the decline of lung function in patients, and is a safe and effective rehabilitation method. Due to the low quality of the included studies, the conclusions of the studies need to be validated by more high-quality randomised controlled trials. | One study suggested that no adverse effects occurred during the study, and the remaining 17 studies were all NR for adverse effects |
| P. Gao2021 | FEV_1_、FEV_1_%、FVC、FEV_1_/FVC%、FEV_1_/pred、quality of life | | Wuqinxi vs control：  ①FEV1[MD=0.39(0.21,0.57),*P*<0.0001,*I^2^* = 73%],4 trials,258  ③FEV1/FVC%[MD=10.39(5.44,15.35),*P*<0.0001,*I^2^* = 97%],8 trials,577  ④FEV1/pred%[MD=8.44,(0.40,16.48),*P*=0.04,*I^2^* = 95%],4 trials,324  ⑤6MWD[MD=63.42(34.06,92.79),*P*<0.0001,*I^2^* = 93%],4 trials,278 | Wuqinxi vs control：  ①FEV1%[MD=4.41(-1.97,10.79),*P*=0.18,*I^2^* = 97%],4 trails,273 | Wuqinxi has an effect on improving lung function in COPD patients, especially FEV1, FEV1/FVC and 6 min walking distance, but large samples and high-quality RCTs are needed to provide more scientific and targeted evidence-based evidence for clinical practice. | NR |
| Xu.S2022 | FEV1、FEV1%、FVC、FEV1/  FVC%、IPmax、EPmax、6MWD、30-s SST、CAT、mMRC/MRC、TCM Symptom Scale、HAMA、HAMD、Self-rated anxiety scale, self-rated depression scale、SGRQ、RR、AR、AC、PaCO_2_、PaO_2_、SpO_2_ | | Lizijue vs control：  ①FEV1[MD=0.17(0.09,0.25),*P*<0.0001,*I*^2^ = 68%],13 trails,936  ②FEV1%[MD=(6.04(3.43,8.65),*P*<0.00001,*I*^2^ = 69%],16 trails,904  ③FEV1/FVC%[MD=6.95(3.06,10.83),*I*^2^ = 95%],19 trails,1186  ④FVC [MD = 0.02（-0.24 ,-0.29), *I*^2^ = 61%],4 trails,249  ⑤EPmax[MD = 1.67( 0.77,2.58), *I*^2^ = 0%],4 trails,247  ⑥IPmax [MD = 1.46(− 0.45,- 3.37), *I*^2^ = 76%],4 trails,247  ⑤6MWD[MD=33.06(23.73,42.38),*I*^2^ =78%],17 trails,1297  ⑥30-s SST[MD=2.65(0.98,4.32),*I*^2^ = 0%],2 trails,69  ⑦CAT[MD=-2.04(-2.77,-1.30),*I*^2^ = 55%],7 trails,554  ⑧mMRC[MD=-0.34(-0.48,-0.20),*I*^2^ = 55%],9 trails,974  ⑨MRC[MD=-0.37(-0.57,-0.18),P=0.0002,*I*^2^ = 15%,],2 trails,134  ⑩TCM Symptom Scale[MD=-1.85(-2.86至-0.85),*I*^2^ = 94%],7 trails,529  ⑪HAMA[MD=-2.31(-3.04至-1.59),*I*^2^ = 58%],4 trails,284  ⑫HAMD[MD=-2.08(-2.45至-1.71),*I*^2^ = 19%],4 trails,284  ⑬SGRQ-total[MD=-6.94(-9.20至-4.67),*I*^2^ = 82%],12 trails,938  ⑭AC [MD = 9.82(4.99–14.65, *I*^2^ = 0%], 3 trails,256  ⑮PaO_2_ [MD = 15.14,(10.60–19.67, *I*^2^ = 54%,] 2 trails,144  ⑯PaCO_2_ (MD = − 1.51( − 2.55 to − 0.46,)*I*^2^ = 92%, 4 trails,420  ⑰RR[MD = 1.43(− 0.53 to 3.39), *I*^2^ = 97%,),4 trails,680  ⑱AR[MD = − 0.91(− 22.99 to 21.17), *I*^2^ = 0%],4 trails,313  ⑲SPO_2_: MD = 0.65( − 1.83 to 3.13, *I*^2^ = 98%],5 trails,764 |  | Lizijue has an adjuvant therapeutic effect on improving lung function, exercise capacity, health status, mental state and quality of life in patients with COPD. | NR |
