# Supplementary material for: Traditional Chinese exercise in chronic obstructive pulmonary disease: An overview of systematic reviews
Source: Medicine (Baltimore). 2024 Jun 28;103(26):e38700. doi: 10.1097/MD.0000000000038700 (PMC11466204; doi:10.1097/MD.0000000000038700)
Supplement: Supplementary file 2 [file medi-103-e38700-s002.docx]

| Supplementary Table 2 SR original study overlap matrix for TCE in COPD | | | | | | | | | | | | | | | | | | |
| --- | --- | --- | --- | --- | --- | --- | --- | --- | --- | --- | --- | --- | --- | --- | --- | --- | --- | --- |
| overlap matrix | **N1** | **N2** | **N3** | **N4** | **N5** | **N6** | **N7** | **N8** | **N9** | **N10** | **N11** | **N12** | **N13** | **N14** | **N15** | **N16** | **N17** | **NO.of times inclued** |
| Chenjinxiu2008 | Y | Y |  |  |  |  |  |  |  |  |  |  |  | Y |  | Y | Y | 5 |
| Zhangwenxia2009 | Y | Y |  |  |  |  |  |  |  |  |  | Y |  | Y |  | Y | Y | 6 |
| chenjinxiu2009a | Y |  |  |  |  |  |  |  |  |  |  |  |  |  |  | Y |  | 2 |
| chenjinxiu2009b | Y |  |  |  |  |  |  |  |  |  |  |  |  |  |  |  | Y | 2 |
| wangzhenwei2010 | Y | Y |  |  |  |  |  |  |  |  |  | Y |  |  |  |  | Y | 4 |
| wangzhenwei2011 | Y |  |  |  |  |  |  |  |  |  |  |  |  |  |  |  | Y | 2 |
| zhaodongxing2011 | Y | Y |  |  |  |  |  |  |  |  |  |  |  |  |  |  |  | 2 |
| fangdongping2012 | Y | Y |  |  |  |  |  |  |  |  |  |  |  |  |  | Y | Y | 4 |
| wangzhenwei2013 | Y |  |  |  |  |  |  |  | Y |  |  | Y |  | Y |  |  | Y | 5 |
| denglijin2009 |  | Y |  |  |  |  |  |  |  |  |  |  |  |  |  |  |  | 1 |
| lidongxiu2011 |  | Y |  |  |  |  |  |  |  |  |  |  |  |  |  | Y |  | 2 |
| zhuzhen2011 |  | Y |  |  |  |  | Y |  |  |  |  | Y |  |  |  | Y |  | 4 |
| baizhonglu2012 |  | Y |  |  |  |  |  |  |  |  |  |  |  |  |  |  | Y | 2 |
| zhuzhen2012 |  | Y |  |  |  |  |  |  |  |  |  |  |  |  |  |  | Y | 2 |
| Bobby2011 |  |  | Y | Y | Y |  |  |  | Y | Y |  |  |  |  |  |  |  | 5 |
| liusurong 2012 |  |  | Y |  |  |  | Y |  |  | Y |  |  |  |  |  |  |  | 3 |
| zhuzhengang 2014 |  |  | Y | Y | Y |  | Y | Y |  | Y | Y |  |  |  |  |  |  | 7 |
| liangxueling 2016 |  |  | Y |  | Y |  |  |  |  | Y | Y |  |  |  |  |  |  | 4 |
| wangjingyu2015 |  |  | Y |  |  |  |  |  |  |  |  |  |  |  |  |  |  | 1 |
| dengyanfang2015 |  |  | Y | Y | Y |  |  |  |  | Y | Y |  |  |  |  |  |  | 5 |
| guoxiujun2016 |  |  | Y |  |  |  |  |  | Y | Y | Y |  |  |  |  |  |  | 4 |
| guojicai2016a |  |  | Y | Y | Y |  | Y | Y |  | Y | Y |  |  |  |  |  |  | 7 |
| chenping2016 |  |  | Y |  |  |  |  |  |  |  | Y |  |  |  |  |  |  | 2 |
| Caocong2016 |  |  |  | Y |  |  |  | Y |  | Y | Y |  |  |  |  |  |  | 4 |
| ChenJinxiu2015 |  |  |  | Y | Y |  |  |  |  |  | Y |  |  |  |  |  |  | 3 |
| ChenYunfeng2015 |  |  |  | Y |  |  |  |  |  | Y | Y |  |  |  |  |  |  | 3 |
| GuoJicai2016b |  |  |  | Y | Y |  | Y | Y |  | Y | Y |  |  |  |  |  |  | 6 |
| LiuSurong2013 |  |  |  | Y | Y |  | Y | Y |  |  | Y |  |  |  |  |  |  | 5 |
| LiuSurong2014 |  |  |  | Y |  |  | Y |  |  | Y | Y |  |  |  |  |  |  | 4 |
| SunYiping2014 |  |  |  | Y | Y |  |  | Y |  | Y |  |  |  |  |  |  |  | 4 |
| YeXinping2016 |  |  |  | Y |  |  |  | Y |  | Y | Y |  |  |  |  |  |  | 4 |
| ZhangLixiu2017 |  |  |  |  | Y |  |  |  |  | Y | Y |  |  |  |  |  |  | 3 |
| Yin2013 |  |  |  |  | Y |  |  |  |  |  |  |  |  |  |  |  |  | 1 |
| HouDafan2017 |  |  |  |  | Y |  |  |  |  | Y | Y | Y |  |  |  |  |  | 4 |
| LiuYunfeng2015 |  |  |  |  | Y |  | Y |  |  |  |  |  |  |  |  |  |  | 2 |
| HuangBaoju2017 |  |  |  |  | Y |  | Y |  |  | Y | Y |  |  |  |  |  |  | 4 |
| WangLi2018 |  |  |  |  | Y |  |  |  |  | Y |  |  |  |  |  |  |  | 2 |
| PanMeiying2016 |  |  |  |  | Y |  | Y |  |  | Y | Y |  |  |  |  |  |  | 4 |
| WangJingyu2015 |  |  |  |  | Y |  |  |  |  |  | Y |  |  |  |  |  |  | 2 |
| FengYichong2009 |  |  |  |  | Y |  | Y | Y |  | Y |  |  |  |  |  |  |  | 4 |
| LuYan2015 |  |  |  |  | Y |  |  | Y |  | Y |  |  |  |  |  |  |  | 3 |
| LiuXiaodan2012 |  |  |  |  | Y |  |  |  | Y |  |  |  |  |  |  |  |  | 2 |
| ChengYufeng2015 |  |  |  |  |  | Y |  |  |  |  |  |  |  |  |  |  |  | 1 |
| HeRui2015 |  |  |  |  |  | Y |  |  |  |  |  |  |  |  |  |  |  | 1 |
| GaoYanfang２０１7 |  |  |  |  |  | Y |  |  |  |  |  |  |  |  | Y |  |  | 2 |
| TanPeihua2016 |  |  |  |  |  | Y |  |  |  |  |  |  |  |  |  |  |  | 1 |
| WeiShanshan2015 |  |  |  |  |  | Y |  |  |  |  |  |  |  |  | Y |  |  | 2 |
| Xing Y.P2017 |  |  |  |  |  | Y |  |  |  |  |  |  |  |  |  |  |  | 1 |
| ZhaoQiaoliang2015 |  |  |  |  |  | Y |  |  |  |  |  |  |  |  | Y |  |  | 2 |
| Zhu.Y 2016 |  |  |  |  |  | Y |  |  |  |  |  |  |  |  |  |  |  | 1 |
| XuYuanhong2010 |  |  |  |  |  |  | Y |  | Y |  |  |  |  |  |  |  |  | 2 |
| LiuXiaodan2011 |  |  |  |  |  |  | Y |  |  |  |  |  |  |  |  |  |  | 1 |
| GuoMingrui2013 |  |  |  |  |  |  | Y |  |  |  |  |  |  |  |  |  |  | 1 |
| DengYanfang2014 |  |  |  |  |  |  | Y | Y |  | Y |  |  |  |  |  |  |  | 3 |
| XueGuangwei 2014 |  |  |  |  |  |  | Y |  |  | Y |  |  |  |  |  |  |  | 2 |
| GaoYing2016 |  |  |  |  |  |  | Y |  |  |  |  |  |  |  |  |  |  | 1 |
| ZhangMin2016 |  |  |  |  |  |  | Y |  |  |  |  |  |  |  |  |  |  | 1 |
| SunYiping 2016 |  |  |  |  |  |  | Y |  |  |  |  |  |  |  |  |  |  | 1 |
| YangBiying2016 |  |  |  |  |  |  | Y |  |  | Y | Y |  |  |  |  |  |  | 3 |
| QiuWenfei2015 |  |  |  |  |  |  |  | Y |  | Y |  |  |  |  |  |  |  | 2 |
| XueGuangwei2015 |  |  |  |  |  |  |  | Y | Y |  |  |  |  |  |  |  |  | 2 |
| [Chun-Mei Xiao](https://pubmed.ncbi.nlm.nih.gov/?term=Xiao+CM&cauthor_id=26131612)2015 |  |  |  |  |  |  |  |  | Y |  |  |  |  | Y |  |  | Y | 3 |
| Zhang M2016 |  |  |  |  |  |  |  |  | Y |  |  |  |  |  |  |  |  | 1 |
| Ge Y2013 |  |  |  |  |  |  |  |  | Y |  |  |  |  |  |  |  |  | 1 |
| Ying G2015 |  |  |  |  |  |  |  |  | Y |  |  |  |  |  |  |  |  | 1 |
| ChenYi2017 |  |  |  |  |  |  |  |  |  | Y | Y |  |  |  |  |  |  | 2 |
| ZhangHuiling2016 |  |  |  |  |  |  |  |  |  |  | Y |  |  |  |  |  |  | 1 |
| ZhuZhenggang 2016 |  |  |  |  |  |  |  |  |  |  | Y |  |  |  |  |  |  | 1 |
| ZhuZhenggang 2017 |  |  |  |  |  |  |  |  |  |  | Y |  |  |  |  |  |  | 1 |
| HuangFang2016 |  |  |  |  |  |  |  |  |  |  | Y |  |  |  |  |  |  | 1 |
| HuangYuanyuan2013 |  |  |  |  |  |  |  |  |  |  | Y |  |  |  |  |  |  | 1 |
| Dai 2018 |  |  |  |  |  |  |  |  |  | Y |  |  |  |  |  |  |  | 1 |
| Li et al2018 |  |  |  |  |  |  |  |  |  | Y |  |  |  |  |  |  |  | 1 |
| Zhang et al2019 |  |  |  |  |  |  |  |  |  | Y |  |  |  |  |  |  |  | 1 |
| Qian et al  2019 |  |  |  |  |  |  |  |  |  | Y |  |  |  |  |  |  |  | 1 |
| Yu et al  2019 |  |  |  |  |  |  |  |  |  | Y |  |  |  |  |  |  |  | 1 |
| Wang 2019 |  |  |  |  |  |  |  |  |  | Y |  |  |  |  |  |  |  | **1** |
| Gu R2017 |  |  |  |  |  |  |  |  |  |  |  | Y |  |  |  |  | Y | **2** |
| Jiang2017 |  |  |  |  |  |  |  |  |  |  |  | Y |  |  |  |  |  | **1** |
| LanYang2016 |  |  |  |  |  |  |  |  |  |  |  | Y | Y | Y |  | Y | Y | **4** |
| Liu Sj 2018 |  |  |  |  |  |  |  |  |  |  |  | Y |  |  |  |  | Y | **2** |
| ShenQian2017a |  |  |  |  |  |  |  |  |  |  |  | Y |  | **Y** |  |  | Y | **3** |
| ShenQian2017b |  |  |  |  |  |  |  |  |  |  |  | Y | **Y** |  |  |  | Y | **3** |
| WUWeibing2018 |  |  |  |  |  |  |  |  |  |  |  | Y |  | **Y** |  | **Y** | Y | **4** |
| ZhaoDongxing2012 |  |  |  |  |  |  |  |  |  |  |  | Y |  | **Y** |  |  | Y | **3** |
| ZhengYingying2015 |  |  |  |  |  |  |  |  |  |  |  | Y |  | **Y** |  |  |  | **2** |
| LiHongqiang2018 |  |  |  |  |  |  |  |  |  |  |  |  | **Y** |  |  |  |  | **1** |
| ZhangFengrui2019 |  |  |  |  |  |  |  |  |  |  |  |  | **Y** |  |  |  | **Y** | **2** |
| ShiJinghui2019 |  |  |  |  |  |  |  |  |  |  |  |  | **Y** | **Y** |  |  | **Y** | **3** |
| ZhangJia2017 |  |  |  |  |  |  |  |  |  |  |  |  | **Y** |  |  |  | **Y** | **2** |
| ChenHongying2017 |  |  |  |  |  |  |  |  |  |  |  |  | **Y** |  |  |  |  | **1** |
| HouMinyan2017 |  |  |  |  |  |  |  |  |  |  |  |  | **Y** | **Y** |  |  | **Y** | **3** |
| JiSiqin2017 |  |  |  |  |  |  |  |  |  |  |  |  | **Y** | **Y** |  |  |  | **2** |
| JuJunfang2019 |  |  |  |  |  |  |  |  |  |  |  |  | **Y** |  |  |  |  |  |
| QuLingling2019 |  |  |  |  |  |  |  |  |  |  |  |  | **Y** |  |  |  | **Y** | **2** |
| LiuTairong2018 |  |  |  |  |  |  |  |  |  |  |  |  | **Y** |  |  |  | **Y** | **2** |
| WU Weibing2018b |  |  |  |  |  |  |  |  |  |  |  |  |  | **Y** |  |  | Y | **2** |
| LI Peijun2018 |  |  |  |  |  |  |  |  |  |  |  |  |  | **Y** |  |  |  | **1** |
| LiRong2018 |  |  |  |  |  |  |  |  |  |  |  |  |  | **Y** |  | **Y** |  | **2** |
| LiuLili2015 |  |  |  |  |  |  |  |  |  |  |  |  |  | **Y** |  |  |  | **1** |
| WangLongbing2015 |  |  |  |  |  |  |  |  |  |  |  |  |  | **Y** |  | **Y** |  | **2** |
| DengLijin 2018 |  |  |  |  |  |  |  |  |  |  |  |  | **Y** | **Y** |  | **Y** | **Y** | **3** |
| ChenZhuo2020 |  |  |  |  |  |  |  |  |  |  |  |  |  |  | **Y** |  |  | **1** |
| LiuFengying2020 |  |  |  |  |  |  |  |  |  |  |  |  |  |  | **Y** |  |  | **1** |
| WangXiaoyan2020 |  |  |  |  |  |  |  |  |  |  |  |  |  |  | **Y** |  |  | **1** |
| ZhuYi2010 |  |  |  |  |  |  |  |  |  |  |  |  |  |  | **Y** |  | **Y** | **2** |
| WangDawei2020 |  |  |  |  |  |  |  |  |  |  |  |  |  |  | **Y** |  |  | **1** |
| ZhangMM 2019 |  |  |  |  |  |  |  |  |  |  |  |  |  |  |  | **Y** | **Y** | **2** |
| Sun N2019 |  |  |  |  |  |  |  |  |  |  |  |  |  |  |  | **Y** | **Y** | **2** |
| Jiang MN 2017 |  |  |  |  |  |  |  |  |  |  |  |  |  |  |  | **Y** |  | **1** |
| Chen FX 2015 |  |  |  |  |  |  |  |  |  |  |  |  |  |  |  | **Y** |  | **1** |
| He JF 2019 |  |  |  |  |  |  |  |  |  |  |  |  |  |  |  | **Y** |  | **1** |
| Li DX2013 |  |  |  |  |  |  |  |  |  |  |  |  |  |  |  |  | **Y** | **1** |
| Ji YW2014 |  |  |  |  |  |  |  |  |  |  |  |  |  |  |  |  | **Y** | **1** |
| Wang2014 |  |  |  |  |  |  |  |  |  |  |  |  |  |  |  |  | **Y** | **1** |
| Guan FG2015 |  |  |  |  |  |  |  |  |  |  |  |  |  |  |  |  | **Y** | **1** |
| Chen HY2016 |  |  |  |  |  |  |  |  |  |  |  |  |  |  |  |  | **Y** | **1** |
| Li p2018 |  |  |  |  |  |  |  |  |  |  |  |  |  |  |  |  | **Y** | **1** |
| Li hq2018 |  |  |  |  |  |  |  |  |  |  |  |  |  |  |  |  | **Y** | **1** |
| Ding MJ2018 |  |  |  |  |  |  |  |  |  |  |  |  |  |  |  |  | **Y** | **1** |
| Zhao JJ2018 |  |  |  |  |  |  |  |  |  |  |  |  |  |  |  |  | **Y** | **1** |
| Ji SQ2019 |  |  |  |  |  |  |  |  |  |  |  |  |  |  |  |  | **Y** | **1** |
| Zhu J2019 |  |  |  |  |  |  |  |  |  |  |  |  |  |  |  |  | **Y** | **1** |
| Shi XL2020 |  |  |  |  |  |  |  |  |  |  |  |  |  |  |  |  | **Y** | **1** |
| **Total studies inclued** | **9** | **10** | **9** | **12** | **20** | **8** | **20** | **12** | **10** | **31** | **25** | **14** | **13** | **18** | **8** | **16** | **40** | **275** |
