# Supplementary material for: Traditional Chinese exercise in chronic obstructive pulmonary disease: An overview of systematic reviews
Source: Medicine (Baltimore). 2024 Jun 28;103(26):e38700. doi: 10.1097/MD.0000000000038700 (PMC11466204; doi:10.1097/MD.0000000000038700)
Supplement: Supplementary file 3 [file medi-103-e38700-s003.docx]

| **Supplementary Table 3 AMSTAR-2 methodologic quality of included systematic reviews** | | | | | | | | | | | | | | | | | |
| --- | --- | --- | --- | --- | --- | --- | --- | --- | --- | --- | --- | --- | --- | --- | --- | --- | --- |
| Author | Item 1 | 2* | 3 | 4* | 5 | 6 | 7* | 8 | 9* | 10 | 11* | 12 | 13* | 14 | 15* | 16 | AMSTAR |
| ZhuangFengkun2015 | Yes | No | No | Yes | Yes | Yes | No | Yes | Yes | No | Yes | No | Yes | No | No | No | Critically low |
| LiuXiaohui2015 | Yes | No | No | Yes | Yes | No | No | Yes | Yes | No | Yes | No | Yes | No | No | No | Critically low |
| HanYan2017 | Yes | No | No | Yes | Yes | No | No | partial Yes | Yes | No | Yes | No | Yes | No | No | Yes | Critically low |
| Lihong2017 | Yes | No | No | Yes | Yes | Yes | No | partial Yes | Yes | No | Yes | No | Yes | Yes | Yes | No | Critically low |
| S.J.Liu2018 | Yes | No | No | partial Yes | Yes | Yes | No | Yes | partial Yes | No | Yes | No | Yes | No | Yes | Yes | Critically low |
| K.Wang2018 | Yes | No | No | partial Yes | Yes | Yes | No | Yes | partial Yes | No | Yes | No | Yes | No | Yes | Yes | Critically low |
| LiJiqiang2018 | Yes | No | No | partial Yes | Yes | Yes | No | Yes | Yes | No | Yes | No | Yes | No | No | Yes | Critically low |
| ChenYanhua2018 | Yes | No | No | partial Yes | Yes | Yes | No | Yes | Yes | No | Yes | No | Yes | Yes | Yes | No | Critically low |
| H.Tong2019 | Yes | No | No | partial Yes | Yes | Yes | Yes | Yes | Yes | No | Yes | No | No | Yes | No | Yes | Critically low |
| A.Cao2020 | Yes | No | No | partial Yes | Yes | Yes | No | Yes | Yes | No | Yes | No | Yes | No | Yes | Yes | Critically low |
| XieQiurong2020 | Yes | No | No | Yes | Yes | No | No | Yes | partial Yes | No | Yes | No | Yes | Yes | Yes | No | Critically low |
| L.Xiao2020 | Yes | Yes | No | partial Yes | No | Yes | No | Yes | Yes | No | Yes | No | Yes | Yes | No | Yes | Critically low |
| LuFeng2021 | Yes | No | No | Yes | No | Yes | No | Yes | partial Yes | Yes | Yes | No | Yes | No | No | No | Critically low |
| ZhangYaqing2021 | Yes | No | No | partial Yes | Yes | Yes | No | Yes | Yes | Yes | Yes | No | Yes | Yes | No | No | Critically low |
| YuanLei2021 | Yes | No | No | partial Yes | Yes | Yes | No | Yes | Yes | No | Yes | No | Yes | Yes | No | No | Critically low |
| P. Gao2021 | Yes | Yes | No | Yes | No | Yes | No | Yes | Yes | Yes | Yes | No | Yes | Yes | Yes | Yes | LOW |
| Xu.S2022 | Yes | No | No | Yes | Yes | Yes | No | Yes | Yes | Yes | Yes | No | Yes | Yes | No | No | Critically low |
| Item1: Did the research questions and inclusion criteria for thereview include the components of PICO?; Item2: Did the report of the review contain an explicit statementthat the review methods were established prior to the conduct ofthe review and did the report justify any significant deviationsfrom the protocol?; Item3: Did the review authors explain their selection of the studydesigns for inclusion in the review?; Item4: Did the review authors use a comprehensive literature searchstrategy?; Item5: Did the review authors perform study selection in duplicate?; Item6: Did the review authors perform data extraction in duplicate?; Item7: Did the review authors provide a list of excluded studies andjustify the exclusions?; Item8: Did the review authors describe the included studies inadequate detail?; Item9: Did the review authors use a satisfactory technique forassessing the risk of bias (RoB) in individual studies that wereincluded in the review?; Item10: Did the review authors report on the sources of funding forthe studies included in the review?; Item11: If meta-analysis was performed, did the review authors useappropriate methods for statistical combination of results?; Item12: If meta-analysis was performed, did the review authorsassess the potential impact of RoB in individual studies on theresults of the meta-analysis or other evidence synthesis?; Item13: Did the review authors account for RoB in primary studieswhen interpreting/discussing the results of the review?; Item14: Did the review authors provide a satisfactory explanationfor, and discussion of, any heterogeneity observed in theresults of the review?; Item15: If they performed quantitative synthesis did the reviewauthors carry out an adequate investigation of publication bias(small study bias) and discuss its likely impact on the resultsof the review?; Item16: Did the review authors report any potential sources ofconflict of interest, including any funding they received forconducting the review?  *:critical item ; #:High:No or one non-critical weakness; Moderate:more than one non-critical weakness; Low:One critical flaw with or without non-critical weaknesses; Critically low:More than one critical flaw with or without non-critical weaknesses. | | | | | | | | | | | | | | | | | |
|  | | | | | | | | | | | | | | | | | |
