# Supplementary material for: Traditional Chinese exercise in chronic obstructive pulmonary disease: An overview of systematic reviews
Source: Medicine (Baltimore). 2024 Jun 28;103(26):e38700. doi: 10.1097/MD.0000000000038700 (PMC11466204; doi:10.1097/MD.0000000000038700)
Supplement: Supplementary file 4 [file medi-103-e38700-s004.docx]

| **Supplementary Table 4 GRADE Quality assessment** | | | | | | | | |
| --- | --- | --- | --- | --- | --- | --- | --- | --- |
| Outcomes |  | Author(year) | Risk of bias | Inconsistency | Indirectness | Imprecision | Publication bias | Overall  quality of evidence |
| Lung function | FEV1 | ZhuangFengkun2015 | -1^①^ | 0 | 0 | 0 | -1^⑤^ | Low |
|  |  | LiuXiaohui2015 | -1^①^ | 0 | 0 | 0 | -1^④^ | Low |
|  |  | HanYan2017 | -1^①^ | -1^②^ | 0 | 0 | -1^④^ | Very low |
|  |  | LiHong2017 | -1^①^ | 0 | 0 | 0 | 0 | Moderate |
|  |  | S.J.Liu2018 | -1^①^ | 0 | 0 | 0 | 0 | Moderate |
|  |  | K.Wang2018 | -1^①^ | 0 | 0 | 0 | 0 | Moderate |
|  |  | ChenYanhua2018 | -1^①^ | 0 | 0 | 0 | -1^④^ | Low |
|  |  | H.Tong2019 | -1^①^ | 0 | 0 | 0 | -1^④^ | Low |
|  |  | A.Cao2020 | -1^①^ | -1^②^ | 0 | 0 | -1^④^ | Very low |
|  |  | XieQiurong2020 | -1^①^ | 0 | 0 | 0 | -1^④^ | Low |
|  |  | L.Xiao2020 | -1^①^ | 0 | 0 | 0 | -1^④^ | Low |
|  |  | YuanLei2021 | -1^①^ | -1^②^ | 0 | 0 | -1^④^ | Very low |
|  |  | P. Gao2021 | -1^①^ | 0 | 0 | 0 | 0 | Moderate |
|  |  | Xu.S2022 | -1^①^ | 0 | 0 | 0 | -1^⑤^ | Low |
|  | FVC | HanYan2017 | -1^①^ | 0 | 0 | 0 | -1^⑤^ | Low |
|  |  | LiHong2017 | -1^①^ | 0 | 0 | 0 | -1^⑤^ | Low |
|  |  | S.J.Liu2018 | -1^①^ | 0 | 0 | 0 | 0 | Moderate |
|  |  | ChenYanhua2018 | -1^①^ | 0 | 0 | 0 | -1^④^ | Low |
|  |  | A.Cao2020 | -1^①^ | 0 | 0 | 0 | -1^④^ | Low |
|  |  | XieQiurong2020 | -1^①^ | 0 | 0 | 0 | -1^④^ | Low |
|  |  | Xu.S2022 | -1^①^ | 0 | 0 | 0 | -1^⑤^ | Low |
|  | FEVl% | ZhuangFengkun2015 | -1^①^ | 0 | 0 | -1^③^ | -1^⑤^ | Very low |
|  |  | LiuXiaohui2015 | -1^①^ | 0 | 0 | 0 | -1^④^ | Low |
|  |  | HanYan2017 | -1^①^ | 0 | 0 | 0 | -1^④^ | Low |
|  |  | LiHong2017 | -1^①^ | 0 | 0 | 0 | -1^④^ | Low |
|  |  | S.J.Liu2018 | -1^①^ | 0 | 0 | 0 | 0 | Moderate |
|  |  | K.Wang2018 | -1^①^ | 0 | 0 | 0 | 0 | Moderate |
|  |  | LiJiqiang2018 | -1^①^ | 0 | 0 | 0 | -1^④^ | Low |
|  |  | ChenYanhua2018 | -1^①^ | 0 | 0 | 0 | -1^④^ | Low |
|  |  | H.Tong2019 | -1^①^ | 0 | 0 | 0 | -1^④^ | Low |
|  |  | XieQiurong2020 | -1^①^ | 0 | 0 | 0 | 0 | Moderate |
|  |  | L.Xiao2020 | -1^①^ | 0 | 0 | 0 | -1^④^ | Low |
|  |  | LuFeng2021 | -1^①^ | -1^②^ | 0 | 0 | -1^④^ | Very low |
|  |  | YuanLei2021 | -1^①^ | -1^②^ | 0 | 0 | -1^④^ | Very low |
|  |  | P. Gao2021 | -1^①^ | 0 | 0 | 0 | 0 | Moderate |
|  |  | Xu.S2022 | -1^①^ | 0 | 0 | 0 | -1^⑤^ | Low |
|  | FEV1/FVC% | ZhuangFengkun2015 | -1^①^ | 0 | 0 | -1^③^ | -1^⑤^ | Very low |
|  |  | HanYan2017 | -1^①^ | -1^②^ | 0 | 0 | -1^④^ | Very low |
|  |  | LiHong2017 | -1^①^ | -1^②^ | 0 | 0 | 0 | Low |
|  |  | S.J.Liu2018 | -1^①^ | 0 | 0 | 0 | 0 | Moderate |
|  |  | K.Wang2018 | -1^①^ | 0 | 0 | 0 | 0 | Moderate |
|  |  | LiJiqiang2018 | -1^①^ | 0 | 0 | 0 | -1^④^ | Low |
|  |  | ChenYanhua2018 | -1^①^ | -1^②^ | 0 | 0 | -1^④^ | Very low |
|  |  | H.Tong2019 | -1^①^ | 0 | 0 | 0 | -1^④^ | Low |
|  |  | A.Cao2020 | -1^①^ | -1^②^ | 0 | 0 | -1^④^ | Very low |
|  |  | XieQiurong2020 | -1^①^ | 0 | 0 | 0 | -1^④^ | Low |
|  |  | L.Xiao2020 | -1^①^ | 0 | 0 | 0 | -1^④^ | Low |
|  |  | LuFeng2021 | -1^①^ | -1^②^ | 0 | 0 | -1^④^ | Very low |
|  |  | YuanLei2021 | -1^①^ | -1^②^ | 0 | 0 | -1^④^ | Very low |
|  |  | P. Gao2021 | -1^①^ | 0 | 0 | 0 | -1^⑤^ | Low |
|  |  | Xu.S2022 | -1^①^ | -1^②^ | 0 | 0 | -1^④^ | Very low |
| exercise endurance | 6MWD | ZhuangFengkun2015 | -1^①^ | 0 | 0 | 0 | -1^⑤^ | Low |
|  |  | LiuXiaohui2015 | -1^①^ | 0 | 0 | 0 | -1^④^ | Low |
|  |  | HanYan2017 | -1^①^ | 0 | 0 | 0 | -1^⑤^ | Low |
|  |  | LiHong2017 | -1^①^ | 0 | 0 | 0 | 0 | Moderate |
|  |  | S.J.Liu2018 | -1^①^ | 0 | 0 | 0 | 0 | Moderate |
|  |  | K.Wang2018 | -1^①^ | -1^②^ | 0 | 0 | 0 | Low |
|  |  | LiJiqiang2018 | -1^①^ | 0 | 0 | 0 | -1^④^ | Low |
|  |  | H.Tong2019 | -1^①^ | 0 | 0 | 0 | -1^④^ | Low |
|  |  | A.Cao2020 | -1^①^ | -1^②^ | 0 | 0 | -1^④^ | Very low |
|  |  | XieQiurong2020 | -1^①^ | 0 | 0 | 0 | -1^④^ | Low |
|  |  | L.Xiao2020 | -1^①^ | 0 | 0 | 0 | -1^④^ | Low |
|  |  | LuFeng2021 | -1^①^ | -1^②^ | 0 | 0 | -1^④^ | Very low |
|  |  | ZhangYaqing2021 | -1^①^ | 0 | 0 | 0 | -1^④^ | Low |
|  |  | YuanLei2021 | -1^①^ | -1^②^ | 0 | 0 | -1^④^ | Very low |
|  |  | P. Gao2021 | -1^①^ | 0 | 0 | 0 | 0 | Moderate |
|  |  | Xu.S2022 | -1^①^ | 0 | 0 | 0 | -1^⑤^ | Low |
| quality of life | SGRQ | ZhuangFengkun2015-activity | -1^①^ | -1^②^ | 0 | -1^③^ | -1^⑤^ | Very low |
|  |  | ZhuangFengkun2015-symptom | -1^①^ | 0 | 0 | -1^③^ | -1^⑤^ | Very low |
|  |  | ZhuangFengkun2015-impact | -1^①^ | 0 | 0 | -1^③^ | -1^⑤^ | Very low |
|  |  | ZhuangFengkun2015-total | -1^①^ | -1^②^ | 0 | -1^③^ | -1^⑤^ | Very low |
|  |  | LiuXiaohui2015-activity | -1^①^ | 0 | 0 | -1^③^ | -1^⑤^ | Very low |
|  |  | LiuXiaohui2015-impact | -1^①^ | 0 | 0 | -1^③^ | -1^⑤^ | Very low |
|  |  | LiuXiaohui2015-symptom | -1^①^ | -1^②^ | 0 | -1^③^ | -1^⑤^ | Very low |
|  |  | A.Cao2020 | -1^①^ | 0 | 0 | 0 | -1^⑤^ | Low |
|  |  | XieQiurong2020 | -1^①^ | 0 | 0 | 0 | -1^⑤^ | Low |
|  |  | L.Xiao2020 | -1^①^ | 0 | 0 | 0 | -1^④^ | Low |
|  |  | LuFeng2021 | -1^①^ | 0 | 0 | 0 | -1^④^ | Low |
|  |  | Xu.S2022-activity | -1^①^ | -1^②^ | 0 | 0 | -1^⑤^ | Very low |
|  |  | Xu.S2022-symptom | -1^①^ | -1^②^ | 0 | 0 | -1^⑤^ | Very low |
|  |  | Xu.S2020-impact | -1^①^ | -1^②^ | 0 | 0 | -1^⑤^ | Very low |
|  |  | Xu.S2020-total | -1^①^ | 0 | 0 | 0 | -1^⑤^ | Low |
|  | CAT | LiJiqiang2018 | -1^①^ | 0 | 0 | 0 | -1^⑤^ | Low |
|  |  | ChenYanhua2018 | -1^①^ | -1^②^ | 0 | 0 | -1^④^ | Very low |
|  |  | H.Tong2019 | -1^①^ | -1^②^ | 0 | 0 | -1^⑤^ | Very low |
|  |  | A.Cao2020 | -1^①^ | -1^②^ | 0 | 0 | -1^④^ | Very low |
|  |  | XieQiurong2020 | -1^①^ | -1^②^ | 0 | -1^③^ | -1^⑤^ | Very low |
|  |  | L.Xiao2020 | -1^①^ | 0 | 0 | 0 | -1^⑤^ | Low |
|  |  | LuFeng2021 | -1^①^ | 0 | 0 | 0 | -1^④^ | Low |
|  |  | ZhangYaqing2021 | -1^①^ | 0 | 0 | 0 | -1^④^ | Low |
|  |  | Xu.S2022 | -1^①^ | 0 | 0 | 0 | -1^⑤^ | Low |
|  | MRC/mMRC | L.Xiao2020 | -1^①^ | 0 | 0 | 0 | -1^⑤^ | Low |
|  |  | LuFeng2021 | -1^①^ | 0 | 0 | 0 | -1^④^ | Low |
|  |  | ZhangYaqing2021 | -1^①^ | 0 | 0 | 0 | -1^④^ | Low |
|  |  | P. Gao2021 | -1^①^ | 0 | 0 | 0 | 0 | Moderate |
|  |  | Xu.S2022-mMRC | -1^①^ | 0 | 0 | 0 | -1^⑤^ | Low |
|  | TCM Symptom Scale | Xu.S2022-MRC | 0 | 0 | 0 | 0 | -1^⑤^ | Moderate |
|  |  | Xu.S2022 | 0 | 0 | 0 | 0 | -1^⑤^ | Moderate |
| ①There is large bias in the methodology of the included researches；②The overlap of confidence intervals is small, and the heterogeneity I^2^ value is relatively large;③The sample size of the included study is too small and the confidence interval is wide；④Asymmetric funnel chart；⑤The number of included studies is small, or all are positive results. | | | | | | | | |
